# The combined impact of low temperatures and shifting phosphorus availability on the competitive ability of cyanobacteria

**DOI:** 10.1038/s41598-022-20580-2

**Published:** 2022-09-30

**Authors:** Charlotte L. Briddon, Edina Szekeres, Adriana Hegedüs, Maria Nicoară, Cecilia Chiriac, Maria Stockenreiter, Bogdan Drugă

**Affiliations:** 1 Institute of Biological Research (NIRDBS), 48 Republicii Street, 400015 Cluj-Napoca, Romania; 2 Biology Centre of the Czech Academy of Sciences, Institute of Hydrobiology, 37005 České Budějovice, Czech Republic; 3 Department of Biology II, Experimental Aquatic Ecology, Ludwig-Maximilians-Universitӓt Müchen, Groβhaderner Str. 2, 82152 Planegg-Martinsried, Germany

**Keywords:** Ecology, Microbiology

## Abstract

In freshwater systems, cyanobacteria are strong competitors under enhanced temperature and eutrophic conditions. Understanding their adaptive and evolutionary potential to multiple environmental states allows us to accurately predict their response to future conditions. To better understand if the combined impacts of temperature and nutrient limitation could suppress the cyanobacterial blooms, a single strain of *Microcystis aeruginosa* was inoculated into natural phytoplankton communities with different nutrient conditions: oligotrophic, eutrophic and eutrophic with the addition of bentophos. We found that the use of the bentophos treatment causes significant differences in prokaryotic and eukaryotic communities. This resulted in reduced biodiversity among the eukaryotes and a decline in cyanobacterial abundance suggesting phosphorus limitation had a strong impact on the community structure. The low temperature during the experiment lead to the disappearance of *M. aeruginosa* in all treatments and gave other phytoplankton groups a competitive advantage leading to the dominance of the eukaryotic families that have diverse morphologies and nutritional modes. These results show cyanobacteria have a reduced competitive advantage under certain temperature and nutrient limiting conditions and therefore, controlling phosphorus concentrations could be a possible mitigation strategy for managing harmful cyanobacterial blooms in a future warmer climate.

## Introduction

Freshwater environments support nearly 6% of all described species yet only make up 0.01% of the planet’s water and cover approximately 0.8% of the Earth’s surface ^1^. Freshwater lakes provide a rich biodiversity making them an invaluable natural resources in ecological, economic, scientific and cultural terms^2^. However, a rapid expansion of the world’s population and associated increases in industrialisation, land use changes and environmental degradation, has had a detrimental impact on freshwater lakes^3^. Nutrient enrichment, specially of nitrogen (N) and phosphorus (P), both promote eutrophication and cyanobacterial algal blooms (CyanoHABS; one of the few groups able to dominate the entire primary producer community) in freshwater lakes^4,5^. Coincidently, the rapid eutrophication also corresponds with freshwater lakes coming under pressure from climatic drivers, such as increased warming and changes in the hydrological cycle^6^. Over the last century, global warming has become one of the most serious issues facing freshwater environments, with temperatures in temperate European lakes increasing on average by 0.58 °C decade^−1^^7^. Global warming and eutrophication are considered two of the most important causes to the occurrence of cyanobacteria blooms in freshwater aquatic ecosystems^8,9^.

Eutrophication caused by human activities can cause significant shifts in freshwater primary producer communities towards fast growing species such as cyanobacteria resulting in harmful algal blooms^10,11^. Concordantly, unprecedented rates of global warming in recent decades has also led to a rise in the frequency of CyanoHABS in temperate lakes^8,12^ as they exhibit optimal growth rates at temperatures greater than 25 °C^13^ and compete most effectively against eukaryotic algae, resulting in a decline in diversity^9^. Increased temperatures can also exacerbate the problem of bottom water anoxia and internal loading of nutrients especially P, enhancing stratification, which can further fuel CyanoHABs^14^. Therefore, reducing P is an important factor in controlling eutrophication and mitigating algal blooms such as CyanoHABs^15^.One cost-effective approach is the use of bentophos (also known as PhosLock), a patented lanthanum-modified bentonite clay formation which binds with bioavailable P, removing it from the system^16^. Previous studies have shown the ability of bentophos to reduce P in the water column, as an effective measure to manage eutrophication^17^ and to reduce CyanoHABs^18^. Furthermore, Bishop and Richardson^19^ found the use of bentophos caused a shift in cyanobacteria abundance, composition and density whilst Lang et al.^17^ found a relative increase in the proportion of non-cyanobacteria planktonic algae such as cryptophytes, chlorophytes and diatoms. However, there is limited work on how the use of bentophos influences freshwater communities at a genus and species level. Therefore, further research is needed into how it affects freshwater ecosystem and whether it can ‘revert’ them back to a pre-eutrophication state.

A well-known bloom forming cyanobacterial genus is *Microcystis*, a strong competitor against other phytoplankton groups such as chlorophytes, cryptophytes and siliceous algae under elevated nutrients and especially temperature conditions^20^. Generally, it is predicted that cyanobacteria will increase in dominance under climate change^13^, though empirical data on their evolutionary responses are scarce. Although, it appears that cyanobacteria vary in their response to higher temperatures over a wide range of nutrient levels and light intensities^13,21^. Preliminarily mesocosm results suggest that cyanobacteria (specially *Microcystis*) have the strong potential to cope under warming conditions and that previous exposure to warming was a critical factor in the phytoplankton community composition^22^. The use of mesocosm experiments allows for the assessment of the competitive ability of ‘adapted’ species within natural communities over multiple trophic states^23^, especially as recent studies suggest that cyanobacteria may have a reduced competitive advantage under nutrient limiting conditions^24^. The use of a common cyanobacterial species (such as *Microcystis aeruginosa*) adapted to future conditions could help determine how cyanobacteria and other algae groups will react to the use of P remediation strategies such as bentophos. Utilising mesocosms with different nutrient conditions, allows us to directly compare the effects on microbial and algal communities and to test if bentophos can improve the water quality of eutrophic freshwater systems.

This research aims to assess the combined effects of temperature and nutrient limitation on cyanobacterial populations. Firstly, we tested the impact of nutrient limitation using three different environments, (1) oligotrophic, (2) eutrophic and (3) eutrophic with the addition of bentophos on microbial and algal communities to determine if it gave cyanobacteria a competitive advantage against other algae groups. Secondly, we analysed the impact of temperature by using a *M. aeruginosa* strain adapted to ambient (22 °C, mean summer temperature) and a heated temperature (26 °C, the predicted mean temperature by 2100) on natural planktonic communities to determine if further adaptation gives certain cyanobacterial species such as *M. aeruginosa* a competitive advantage. Thirdly, we then compared these results with the mesocosm experiments carried out in 2019^22^ with the same strain of *M. aeruginosa*, to determine if the concordant effects of nutrient limitation and temperature result in different phytoplankton communities.

## Results

### Growth rate evolution

**Figure 1:**
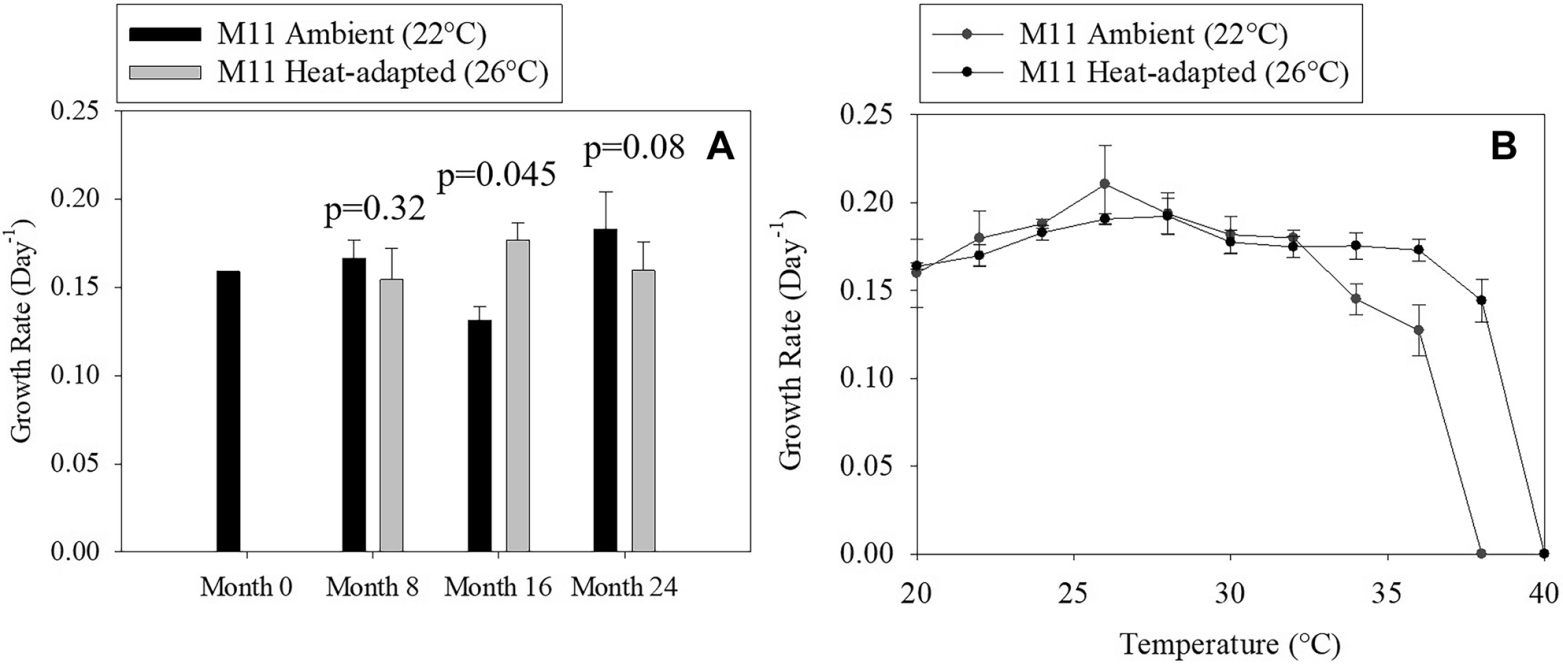
(**A**) Mean Growth rate and standard deviation of *M. aeruginosa* (strain M11) measured every 8 months for ambient (M11_22) and heat-adapted (M11_26) and (**B**) the thermal reaction norms with standard deviation showing the growth rate at 2 °C intervals between 20 and 40 °C for both ambient and heat-adapted M11.

The growth rates of *M. aeruginosa* strain (M11) grown for 2 years at 22 °C (ambient) and 26 °C (heated) were measured in the control environment (22 °C) every 8 months (Months 0, 8, 16 and 24). This was to determine if adaptation resulted in enhanced growth under ambient temperature over time. At Month 0, the M11 growth rates were similar (μ = 0.156–0.159 day^−1^; Fig. 1A). The growth rates for the strain grown at both temperatures fluctuated, showing no pattern after 2 years of exposure, with similar growth rates to those measured at the beginning of the experiment (Month 0). At the end of the experiment (Month 24) the growth rate of the ambient and heat-adapted M11 showed no significant differences in growth rate (as both were measured at 22 °C). The differences between growth rates between the ambient and heat-adapted M11 was statistically significant only for Month 8 (*p* < 0.05).

The TRNs results showed clear differences between the growth rates over a range of temperatures for both ambient and heat-adapted M11 (Fig. 1B). The lethal temperature was 2 °C higher for heat-adapted compared to ambient-adapted M11. The growth rates for ambient-adapted M11 were stable until 32 °C, declining until 37 °C before reaching lethal temperature at 38 °C. The growth rates for heat-adapted M11 were constant until 39 °C before reaching lethal temperature at 40 °C.

### qPCR gene expression

**Figure 2:**
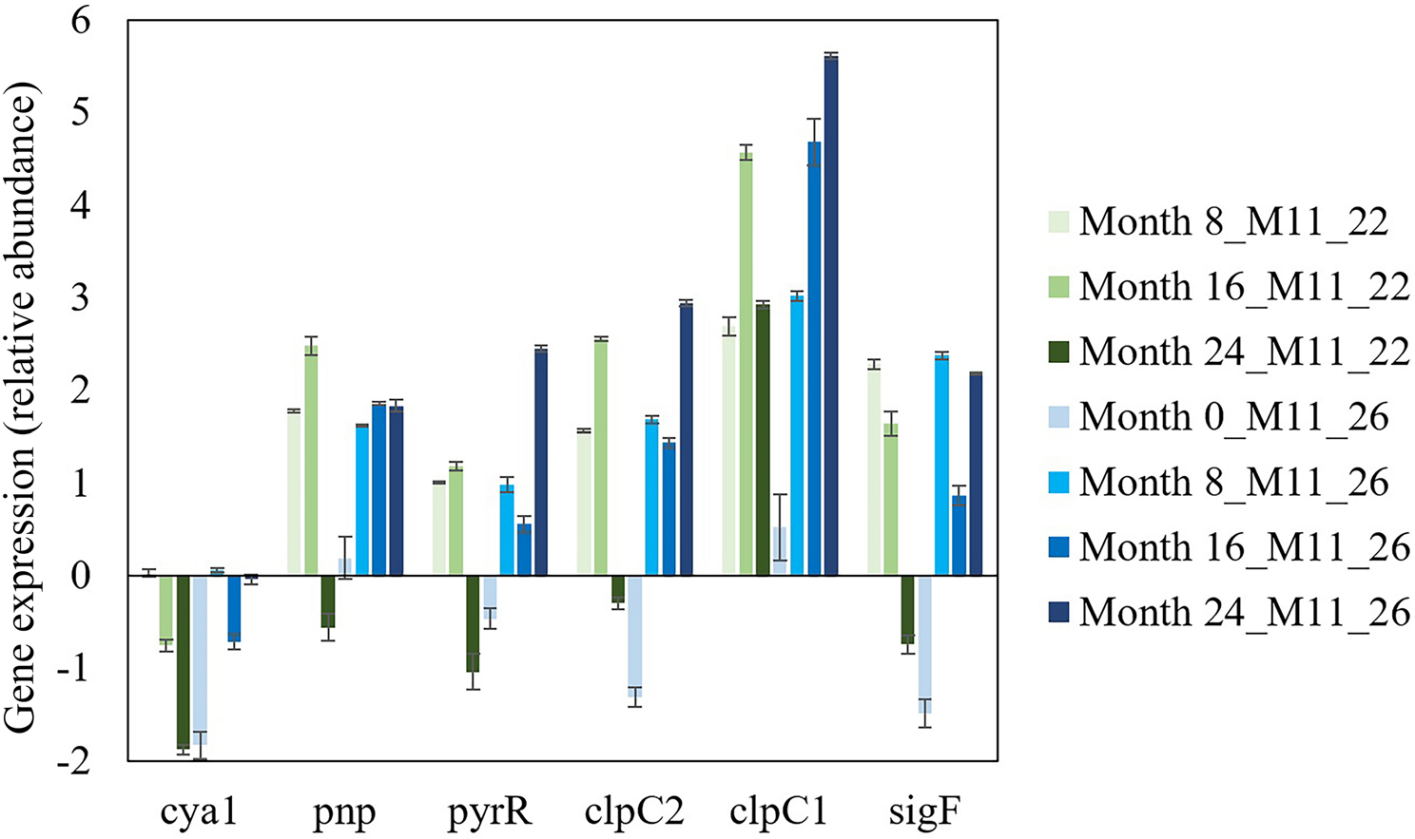
The gene expression results for six genes for M11_22 and M11_26 for Months 0–24. The values represent the relative abundance of target genes based on *rnp*B, which was used as reference gene. These results were normalized against Month 0_M11_22 to determine if temperature has caused a change in gene expression. Each test was done in three replicates.

Candidate genes were selected from different pathways involved in heat shock response or thermal tolerance in cyanobacteria^25^. The qPCR analysis results were calculated as fold change in the expressions of target genes (2^−ΔΔCt^): *cya*1, *pnp*, *pyr*R, *clp*C1, *clp*C2, and *sig*F within the specific time period and samples (Months 0, 8, 16 and 24 for ambient- and heat adapted M11) relative to the *M. aeruginosa* M11 *rnp*B (reference gene). Ambient adapted M11 showed a reduction in gene expression levels with 0.3–1.9-fold reduction by Month 24 (Fig. 2). The expression of *clp*C1showed an up-regulation trend in comparison to all other genes of interest. Heat adapted M11 displayed an overall upregulation over the time period of the experiment, with an approximately 1.8–5.6-fold increase in gene expression of *clp*C1. By Month 24, the largest increase was observed for *clp*C1 gene with a > fivefold gene expression within heat adapted M11, demonstrating a steady increase within this study (Fig. 2). The *cya*1 gene profiles showed the least change of expression for both ambient and heat adapted M11, with a slight down-regulation.

### Land-based mesocosm experiments - temperature and light intensity

The fluctuations in temperature of the three treatments were similar throughout the whole mesocosm experiment. The mean temperature throughout the first 14 days of the experiment averaged c.21–22 °C before declining in all treatments in the third week to 16 °C (Fig. S1A). In the fourth week, the water temperature increased back to an average of c.21–22 °C. The mesocosms with the oligotrophic treatment consistently had a daytime temperature approx. 1 °C higher than the other two treatments. Oligotrophic systems have lower algae abundance and higher light intensity at depth (reflected in the light intensity measurements taken during this study) due to greater water transparency, causing a slightly elevated temperature.

The light intensity measurements were highest in the oligotrophic compared to the other two treatments (Fig. S1B). The light intensity measurements were relatively stable throughout the four-week experiment but there was a slight dip in week 3 (coinciding with the decline in temperature) for all treatments.

### Nutrients

Concentrations for all nutrients were lowest in the oligotrophic treatment (Table S1). The eutrophic treatment had the highest levels of phosphorus (4.20 μgL^−1^ for PO_4_-P; 35.41 μgL^−1^ for TP) while the use of bentophos led to a reduction in P (to 2.87 μgL^−1^ for PO_4_-P; 19.18 μgL^−1^ for TP). The bentophos treatment had the highest concentration of both NO_2_ (16.55 μgL^−1^) and NO_3_^−^ (732.01 μgL^−1^).

### Phytoplankton group dynamics

**Figure 3:**
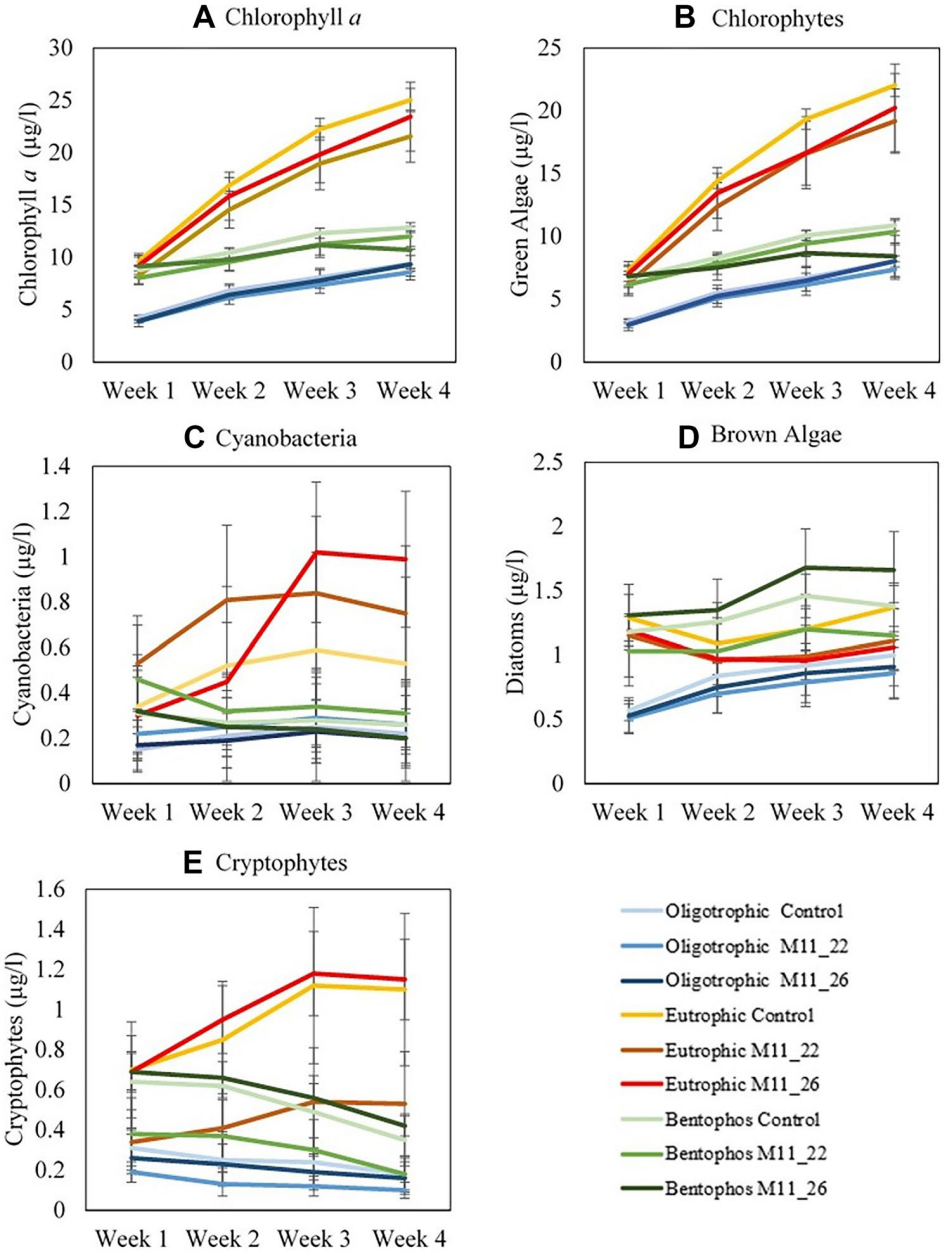
Mean (**A**) Chlorophyll *a*, (**B**) Chlorophyte, (**C**) Cyanobacteria, (**D**) Brown algae and (**E**) Cryptophytes concentrations for each week of the experiment for all treatments. Each test was done in three replicates.

Chlorophyll *a* concentration (µL^-1^) increased as the experiment progressed, with the largest increase seen in the eutrophic treatment and the smallest in the bentophos treatment (Fig. 3). There were significant differences (using ANOVA) between Week 1 and Week 4 concentrations (*p* < 0.001, = 16.637; Table S2). The greatest increase was observed between Week 1 and Week 2 (*p* = 0.014, *F* = 16.637), with a smaller increase but still a significant rise through Weeks 2–4 (*p* = 0.002, *F* = 16.637). The eutrophic treatment showed the largest increase in mean chlorophyll *a* concentrations of 9.04 (Week 1) to 23.34 μgL^−1^ (Week 4). While the oligotrophic and bentophos treatments saw an increase in mean chlorophyll *a* of 4.02–9.10 μgL^−1^ and 8.67–11.85 μgL^−1^ respectively over the same time period. No significant differences or increases were observed in chlorophyll *a* concentration between the control and the mesocosms inoculated with M11_22 and M11_26 (Fig. 3).

The most noteworthy result was that there were no discernible differences between the brown group, cyanobacteria and cryptophyte concentrations throughout the four-week experiment, with chlorophytes showing the only significant change in concentrations (*p* < 0.001). Mean chlorophyte concentration increased significantly in all treatments as the experiment progresses, with the largest increase in the eutrophic mesocosm (6.86–20.48 μgL^−1^; Fig. 3). Mean chlorophyte concentrations increased 4.76 and 3.33 μgL^−1^ for the oligotrophic and bentophos treatments respectively. In general, the groups of microalgae were not affected by the inoculation of ambient or heat-adapted *M. aeruginosa*, with similar composition among all three treatments. The mean cyanobacteria concentration was slightly higher in the eutrophic treatment compared to the other two treatments, with the highest concentration in the mesocosms inoculated with heat-adapted M11, followed by ambient-adapted M11 and then the control (Fig. 3C).

### 16S and 18S metabarcoding data

A total of 1.0 million sequence reads were obtained following the 16S rDNA amplicon sequencing and 1.4 million for the 18S rDNA. A number of 503,557 reads (248,090 for 16S and 255,467 for 18S) passed the processing and filtering steps (sequencing quality and read length). Following reassembly, alignment clean-up and mapping, the final abundance of OTUs from the nine samples contained 520 prokaryotic OTUs and 371 eukaryotic OTUs.

**Figure 4:**
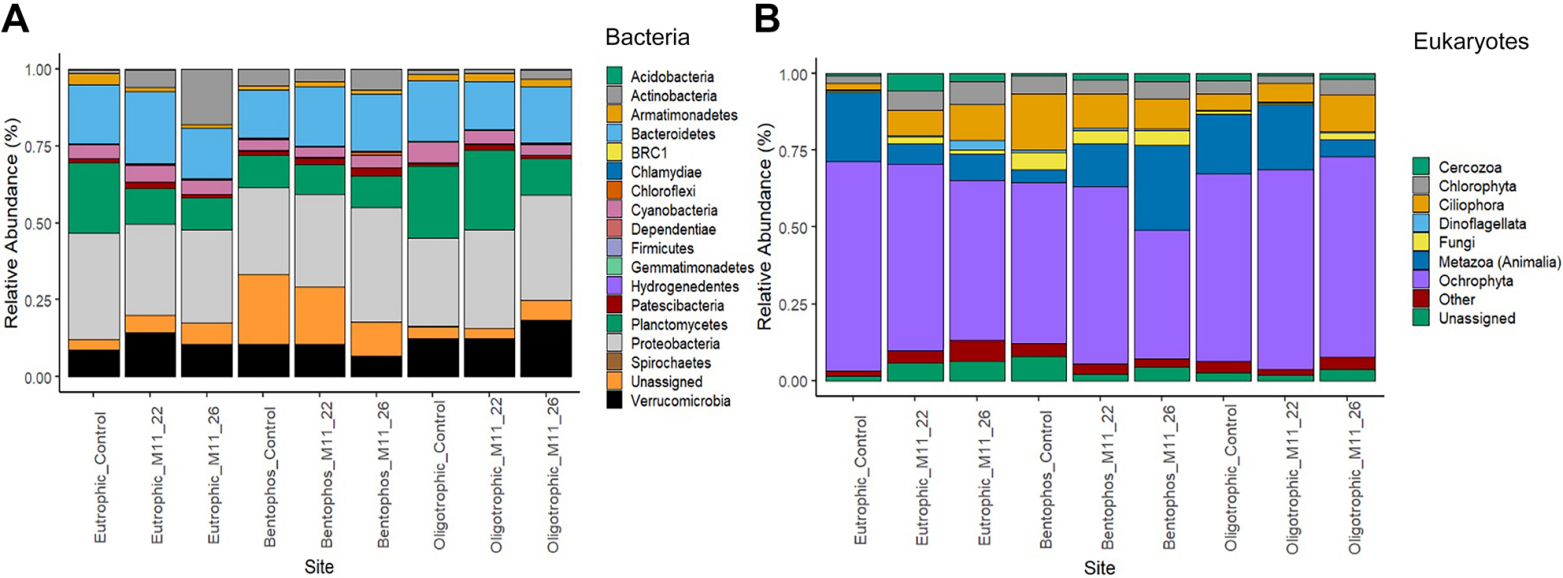
DNA Metabarcoding results to the (**A**) phylum level of the 16S gene for prokaryotes and (**B**) order level of the 18S gene for eukaryotes collected at the end of the four-week experiment. The other category is all orders with < 1% total abundance.

The three treatments had a similar composition of bacterial communities, yet the abundances differed (Fig. 4A; Table S3). The most abundant prokaryotic OTUs identified belong to the phylum Proteobacteria (28.2–37.0%), with a majority of these OTUs assigned to Alphaproteobacteria (22.7–30.5%) and Gammaproteobacteria (3.6–5.6%) (Fig. 4A). The main difference between the three treatments was a lower abundance of Planctomycetes in the bentophos treatment (< 10.7%) compared to the oligotrophic (11.8–25.9%) and the eutrophic treatments (10.3–22.9%), with the majority consisting of the orders Pirellulales and Planctomycetales (Fig. S2). Higher concentrations of Actinobacteria (consisting of 90% Frankiales) were found in the eutrophic treatment inoculated with heat adapted *M. aeruginosa* (17.8%) compared to the samples inoculated with ambient *M. aeruginosa* and the oligotrophic tanks (< 5.8%). There was a high abundance of Bacteroidetes (15.9–23.5%) observed in all samples with no differences between treatments or the strain of *M. aeruginosa*. Verrucomicrobia was observed in higher abundances in the oligotrophic and eutrophic treatments (8.7–18.2%) compared to bentophos treatment (6.7–10.4%).

Cyanobacteria were found in low abundances in all samples (3.3–6.7%), with no differences between the samples inoculated with *M. aeruginosa* and the control samples (Fig. 4A). Even though the samples were inoculated with *M. aeruginosa* (except the control samples)*,* no *Microcystis* was present in any of the samples. Most cyanobacterial DNA reads were assigned to the genus *Pseudanabaena* and *Synechococcus* (Fig. S2), however, due to sequence ambiguity, individual species could not be identified. Chloroflexi (a phylum of thermophilic bacteria) were found in slightly higher abundances in the tanks inoculated with heat adapted *Microcystis* (0.3–1.0%) compared to other tanks (< 0.2%). A higher proportion of the DNA sequences from the bentophos treatment (11.18–22.66%) compared to < 6.99% in the other treatments remained unassigned.

The eukaryotic populations in all samples were dominated by the SAR supergroup (a clade that includes Stramenopiles, Alveolates and Rhizaria;^26^) with abundances of 56.4–80.3% (Fig. 4B; Table S4). The majority of the SAR supergroup consists of the family Ochrophyta 42.0–68.4% of total abundance, followed by Ciliophora (2.3–18.4%) and Cercozoa (0.7–5.6%; Fig. S3). There was no discernable pattern between Ochrophyta, Ciliophora and Cercozoa abundance and a treatment/*Microcystis* inoculation. The second most dominant group was from the order Metazoa consisting mostly of the family Copepoda (0.7–24.9%) followed by Monogonota (0.6–3.1%). A high abundance of copepods was observed in samples Oligotrophic_M11_22, Oligotrophic_Contol, Eutrophic_Control, Bentophos_M11_22 and Bentophos_M11-26 (13.0–24.9%) compared to < 5.1% in the remaining samples (Fig. 4B). Chlorophytes were present in all samples with abundances ranging from 2.3 to 7.4%. Low abundances of fungi (mostly belonging to the order Cryptomycota) were identified in all samples with the highest abundance found in the bentophos treatment. Dinoflagellates were present in low abundances in all samples (< 3.2%). A variable proportion of reads (1.8–7.8%) were unassigned.

**Figure 5:**
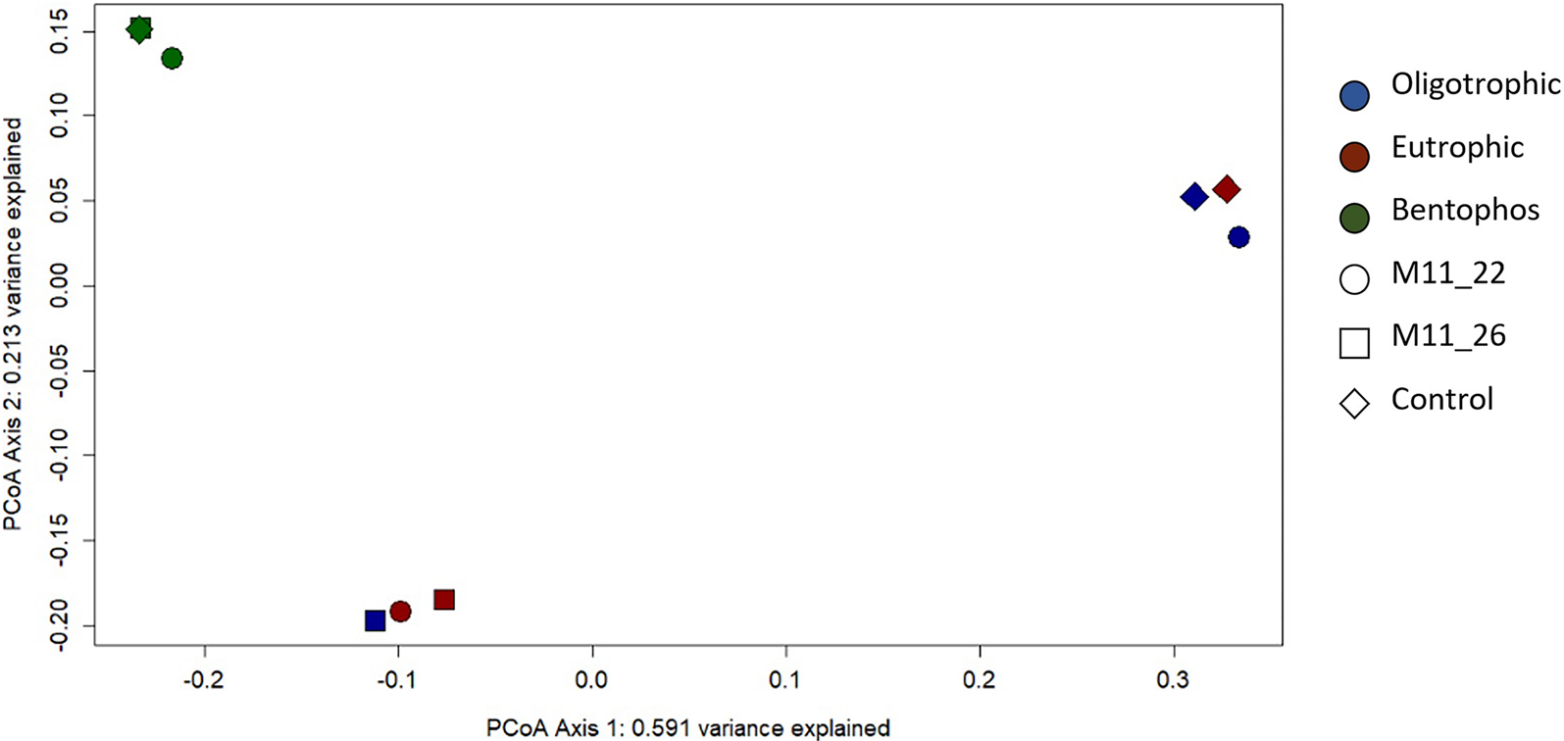
PCoA biplot of the 16S and 18S Bray–Curtis distance matrix for each mesocosm showing a distinct grouping for the bentophos treatment.

The PCoA of the bacterial communities showed a clear difference between the bentophos and the other two treatments (Fig. 5), which were distinguished by higher abundances (compared to the oligotrophic and eutrophic treatments) of bacteria only found in metagenomic data (Hydrogenedentes, Dependentiae, Gemmatimonadetes and Patescibacteria) and a lack of cyanobacterial taxa (Fig. S4). The remaining two groups consisted of a mixture of the oligotrophic and eutrophic treatments characterised by high abundances of Planctomycetes, cyanobacteria, BRC1 and Spirochaetes. The separation of bentophos treatment along both the first and second axis showed the impact of the bentophos on the bacterial communities’ composition (Fig. S4A). Compared to the 16S PCA, there were no clear groupings between treatment or the isolate of M11 used in the 18S PCA due to the dominance of Ocrophyta and Ciliophora (Fig. S4).

The variation in terms of beta diversity (using weighted Unifrac distances) was explained by the treatment specifically shown by the clear grouping of the bentophos treatment, as shown by axis 1 (which explained 59.10% of the variance). There were no other clear groupings for the other two treatments nor between the isolate of M11 used (Fig. 5).

### Comparison with previous experimental mesocosm results

Both the 2019^22^ and 2020 mesocosm experiments had a similar bacterial composition but different abundances. Compared to the 2019 experiment, the 2020 mesocosms displayed a lower abundance of proteobacteria, planctomycetes, and especially cyanobacteria. The latter had a much smaller abundance of 3.3–6.7% compared to 2019 (20–50%). The main difference between the two experiments is the lack of dominance of the SAR supergroup in the 2019 experiment, with the most abundant group being Metazoa consisting of copepods (7–30% of total reads) and gastrotrichs (7–33% of total reads).

## Discussion

The incorporation of long-term adapted *M. aeruginosa* into short-term mesocosm experiments can help explore the mechanisms involved in the adaptation process^27^ and see if increased temperature gives the strain a competitive advantage over natural phytoplankton communities. Analysis of the qPCR gene expression of *M. aeruginosa* showed an increased upregulation of genes associated with enhanced thermal tolerance (Fig. 2). A continuous increase in upregulation in the heat-adapted M11 (but not the ambient-adapted M11) over time of the genes *clp*C1 and *pnp* suggests adaptation to increased temperature ‘improved’ over time. There was also an increase in the expression of *sigF* and *pyrR* genes although this was not proportional to the level of adaption. Tillich et al.^25^ found that the *clp*C1*, pnp, sigF* and *pyrR* genes gave the highest fitness advantage at enhanced temperatures and are important for increased thermal tolerance in *Synechocystis*. These results suggest that the enhanced expression and upregulation of these genes gave heat-adapted M11 an advantage over the ambient-adapted M11in warmer temperatures. This is supported by a former mesocosm experiment ^22^, who found that high temperatures (30 °C) gave heat-adapted *M. aeruginosa* a strong competitive advantage over other algal groups leading to significantly higher concentrations. However, we were not able to replicate these results as low temperatures for the duration of this experiment most likely lead to the disappearance of both ambient and heat adapted *M. aeruginosa.* Nevertheless, we found that the use of bentophos lead to a substantial reduction in cyanobacterial taxa suggesting that shifting P availability could help reduce the group’s competitive ability under warming conditions.

Interestingly, the bentophos treatment showed significant differences from the other two treatments suggesting P limitation has impacted algal and bacterial communities in freshwater lakes (Fig. 5). These samples were characterised by a lack of cyanobacterial taxa (3.3–4.0%) and higher abundances of small celled non-autotroph bacteria only identified in metagenomic data (Hydrogenedentes, Dependentiae, Gemmatimonadetes and Patescibacteria; Fig. S4A). Limited information on the ecology of these bacteria suggests they are generalists, as they have been found widespread across diverse environments^28–30^. Also, Patescibacteria have a PhoR-PhoB two component regulatory system, which is advantageous in low P environments^31^. Moreover, the eukaryotic communities did not show a clear difference between the bentophos and the other treatments, which were characterised by taxa which comprised of < 1% of the relative abundance (*Goniomonas,* choanoflagellida and *Diphylleia rotans*; Fig. S4B). These orders are cosmopolitan species found in a wide range of freshwater environments with varying nutrient regimes^32–34^. Multiple studies have found that the use of bentophos in temperate lakes led to a significant reduction in cyanobacteria and a shift away from cyanobacteria dominance^17,35^. bentophos seems to have a greater impact on cyanobacteria compared to eukaryotic planktonic groups, as N_2_ fixing cyanobacteria (which bloom under N limited conditions, common in eutrophic lakes^36^) have a stronger P requirement^35,37^. This could be a potential reason for the lack of N_2_ fixing cyanobacteria residence populations. It could be suggested that the use of bentophos (which binds to the available P preventing it from being utilised by microalgae and bacteria), altered the N:P ratio (to 38.2; Table S1) leading to P limitation. This could have led to a reduction in cyanobacteria taxa, a rise in generalist small cell bacteria and Patescibacteria) who have developed the ability to survive under low P conditions.

The cyanobacteria taxa in all samples (3.3–6.7%) were dominated by the genera *Synechococcus* and *Pseudanabaena* due to their ability to compete well under nutrient limiting and varying light intensities^38^. Studies from temperate freshwater lakes (Tiefer See, Germany^38^) found a dominance of *Synechococcus* in the late Spring/early Summer due to their physiological conditions such as rapid growth, ability to adapt to changing light conditions and affinity to low chlorophyll *a* conditions^39^. Under nutrient limiting conditions, *Synechococcus* has the ability to utilise orthophosphate and other organic phosphorous sources apart from inorganic phosphates and to store N^40^, enhancing its competitive ability against other phytoplankton groups^41^. Surprisingly the bentophos treatments (2.0–3.1%) did not have the highest relative abundance of *Synechococcus,* with this treatment being characterised by low abundance (Fig. S2). Even though *Synechococcus* has an extreme adaptability to a wide range of temperatures, it has improved growth at higher temperatures^42,43^ suggesting the low temperatures during the experiment dampened their growth. *Synechococcus* success in oligotrophic treatments could be due to its capacity to adapt to high light conditions in oligotrophic environments^43^. Some strains of *Pseudanabaena* spp. have the ability to regulate the ratio of the accessory photosynthetic pigments phycocyanin and phycoerythrin, in response to changing light wavelengths^44^ helping them to adapt to the prevailing light spectrum^45^. This could explain why the oligotrophic treatment with the highest light intensity had the highest abundance of *Pseudanabaena* spp (0.8–5%). Nevertheless, similar abundances in the eutrophic conditions suggest that there must be other contributing factors. Abiotic factors that could explain this include grazing by ciliates and protists, viral lysis and nutrient availability^46,47^.

Surprisingly, even though *M. aeruginosa* were inoculated into all mesocosms (except the controls), it had disappeared by the end of the four-week experiment (Fig. S2). Whereas, in the 2019 mesocosm experiment, *M. aeruginosa* (M11) consisted of 49% OTUs (in ambient tanks) and 36% OTUs in heated tanks^22^. *M. aeruginosa* is a strong competitor under increased temperatures but growth is suppressed when the temperature is < 20 °C^48^. Also, the analysis of temperature-dependent growth rates determined that many eukaryotic algae had maximised or declining growth rates at > 20 °C, whereas the growth rate of many cyanobacteria species increased^13,49^. Previous mesocosm, laboratory and larger-scale limnological studies have also found that *Microcystis* spp. yielded increased growth rates under enhanced temperatures^50–52^. This is further supported by the 2019 mesocosm experiment^22^, which had temperatures up to 32–33 °C, most likely aiding the dominance of *M. aeruginosa*^13^. This suggests that the low temperature (< 22 °C) recorded in the mesocosms could have reduced their competitive ability and limited its growth, resulting in the disappearance of *M. aeruginosa*^53^.

All treatments showed a similar bacterial community composition after the four-week mesocosm experiment (Fig. 4A; Table S5). Proteobacteria, a group of gram-negative bacteria, dominated bacterial populations in all treatments suggesting its relative abundance was not influenced by trophic state or nutrient limitation. Similar relative abundances of proteobacteria were expected as they have been observed in a wide range of freshwater environments (Fig. 4A;^54^). This could also explain why proteobacteria dominated in the 2019 mesocosm despite the different nutrient and temperature conditions^22^. Bacteroidetes was the second most abundant phylum (15.6–23.5%) but there were no significant differences between treatments (Fig. 4A) providing further evidence that this phylum has been found in a diverse range of freshwater habitats^55,56^. There were lower relative abundances of planctomycetes observed in the bentophos treatment (9.5–10.7%) compared to the oligotrophic (11.8–25.9%) and eutrophic (10.3–22.9%) treatments (Fig. 4A). Planctomycetes are particle-associated and mostly grow in nutrient poor oligotrophic environments but have also been found in eutrophic conditions^57^. They have a significant impact on lake biogeochemical cycles through their ability to mineralise and breakdown detritus particles in the water column, making nutrients available to other organisms^29,57^. The reduced availability of nutrients in the oligotrophic treatment (which are characterised by higher abundances of Planctomycetes; Fig. S4A) could also explain the lower abundances in the 2019 mesocosm experiment^22^ and why this treatment also had higher relative abundances of Verrucomicrobia^55,56^. Schmidt et al.^56^ found that in a collection of freshwater lakes this phylum was significantly over-represented in oligotrophic (relative to eutrophic) lakes. Studies have found that temperature and total phosphorus (TP) are the main factors influencing bacterial communities in freshwater lakes^58^. The low and consistent temperatures within all mesocosms (Fig. S1) and the lack of thermophilic prokaryotes such as chloroflexi^59^ suggests that nutrient concentrations including phosphorus are most likely driving the differences between microbial communities in this experiment. Low abundances of *Firmicutes* in all mesocosms (< 0.1%) could be due to aerobic conditions and low cyanobacterial abundances (Fig. 4A), as this family exhibits a significant advantage during the anaerobic decomposition of cyanobacteria blooms^60^. Other phyla occurred in low relative abundances (< 3.0%) probably due to a combination of physical, biological and chemical factors^61^.

The eukaryotic diversity (Fig. 4B; Table S5) in all samples was dominated by the protists from the SAR supergroup specially with the families Orchrophyla, Ciliophora and Cercozoa. The family Orchrophyla were dominated by the genera *Chrysamoeba* and *Ochromonas* whereas the families Ciliophora and Cercozoa had more diverse genera present (Fig. S3). The genera (*Chrysamoeba* and *Ochromonas*) and the family Ciliophora, encompasses a broad range of morphological diversity and a variety of trophic modes within the microbial food web^62^. A likely reason for their dominance is that the species within both genera can have different nutritional modes^63,64^. Generally, mixotrophy enables organisms to overcome unfavourable conditions allowing them to inhibit environments with altered N:P ratios and high light intensities^65^. Mixotrophs have an advantage over heterotrophs under high light intensities, as they can acquire nutrients via phototrophy^66^. Also, mixotrophs have higher cellular N contents (compared to heterotrophs) and therefore a higher demand for N, so are more successful under P limiting conditions^67^. This could explain why there is similar relative abundance of these genera across all treatments regardless of nutrient concentrations or light intensity. Cercozoa is a heterotrophic predatory flagellate of microbacteria and Archea^68^. This family has a high diversity with multiple morphologies such as flagellated (Cercomonadidae, Glissomonadida) and spherical ameboid forms with filopodia (Vampyrellidae; Fig. S3), which could explain its high number of species found across all treatments^69^. Furthermore, lower temperatures for the duration of the experiment most likely aided their competitive ability. Sakharova and Korneva^70^ and Cruaud et al.^71^ found an increase of chrysophytes and Ciliphora during low temperatures in the Rybinsk Reservoir, Moscow and the Saint-Charles River, Canada suggesting these populations were active during low water temperatures. It could be hypothesised that the lower temperatures dampened the competitive ability of other algal groups and combined with nutrient limitation gave the SAR supergroup an advantage over the other phytoplankton groups resulting in their dominance in all treatments.

We observed a decline in both bacterial and eukaryotic species richness compared to the 2019 mesocosm experiment (according to the Shannon Diversity Index; Table S5). The DNA data (based on the PCoA plot; Fig. 5) indicated a significant difference between the bentophos and the other treatments, suggesting nutrient limitation specially of P is the main driver of biodiversity. Only the green algae concentrations showed a significant increase as the experiment progresses for all mesocosms regardless of treatment whilst cyanobacteria, diatom and cryptophytes concentrations either showed insignificant change or a decline in concentration over the four-week experiment (Fig. 3). Lürling et al.^72^ found that the mean growth rates of chlorophytes were significantly higher (0.62 day^−1^) than cyanobacteria (0.42 day^−1^) at 20 °C. This supports previous findings indicating that higher growth rates for chlorophytes under certain temperature regimes^22^. It could be suggested that low temperatures for the duration of this experiment dampened cyanobacterial growth giving chlorophytes a competitive advantage during this experiment. Moreover, other studies have found that chlorophytes dominate in hypertrophic lakes due to the fast growing chlorophytes having a competitive advantage over the slow growing cyanobacteria even when inorganic nutrient concentrations are low^73^. This could explain why the bentophos treatment saw a similar ending relative abundance (according to the metabarcoding data: Fig. 5) to the eutrophic treatment.

## Conclusion

In summary, this experiment has shown that the use of the bentophos treatment causes significant differences in prokaryotic and eukaryotic communities resulting in a decline in cyanobacterial taxa and reduced biodiversity particularly within the eukaryotes. Unseasonably cold temperatures for the duration of the experiment most likely led to the disappearance of the inoculated strain of *M. aeruginosa*, so it was not possible to test how this adapted strain respond under nutrient limiting conditions. It would be interesting to test if the bentophos treatment still has an impact under enhanced temperature and if this causes a reduction in cyanobacteria. Undertaking mesocosms experiments under more favourable weather conditions may help determine if further adaption gives *M. aeruginosa* a competitive advantage under predicted future conditions. This study highlights how boosting the competitive advantage of other groups using bentophos and other P binding materials can be a potential mediation strategy against harmful cyanobacterial blooms.

## Materials and methods

### Biological material and growth conditions of the *Microcystis* strain (M11)

The *M. aeruginosa* strain (M11) used in this study was isolated from Lake Tașaul, Romania (44.35°N 28.61°E), a eutrophic freshwater lake, in the summer of 2018. The lake had an average water temperature of 22 °C. After collection, the samples were deposited as non-axenic cultures in the Collection of Cyanobacteria and Algae (AICB) at the Institute of Biological Research in Cluj-Napoca, Romania^74^. The phylogenetic identity of strain M11 was confirmed using the 16S rDNA gene amplified with specific primers^75^. The PCR fragments were sequenced by a third-party company (Macrogen Europe, Amsterdam, The Netherlands), who confirmed the strain was *M. aeruginosa*.

M11 was adapted for two years (from June 2018 to June 2020) in semi-batch conditions (corresponding to approx. 200 generations) in ultrapure water-based BG11 medium^76^ under controlled 16 h:8 h light:dark conditions provided by white LED lamps (25 μmol photon m^−2^ s^−1^). The strain was grown under two different temperature conditions, 22 °C (mean summer temperature) and 26 °C (the predicted temperature by 2100^77^). Samples were grown in triplicates using 100 ml glass tubes. Each sample was bubbled daily with filtered atmospheric air (0.22 µm; Minisart, Sartorius, Göttingen, Germany) for 90 min (15 min aeration every 4 h). Frequent bubbling was needed to prevent cells gathering and causing self-shading.

### Growth rate measurement and thermal reaction norms (TRNs)

During the two-year adaptation period, the growth rate (μ) of both ambient and heat-adapted M11 were measured at the beginning of the adaptation (June 2020, Month 0) as well as after 8, 16 and 24 months of cultivation at 22 °C and 26 °C. All cultures were tested in their original environment (22 °C) to compare whether long-term acclimatizationoccurred at 26 °C (as compared to 22 °C), to determine whether adapting M11 to 26 °C gave them a competitive advantage when grown under ambient temperature (22 °C) again. Therefore, the growth rate was only measured at 22 °C regardless of whether the strain was previously adapted at 22 °C or 26 °C. Before measuring all cultures were subsampled and diluted to the same starting density of OD_600_ = 0.1 (approximately 1.06 × 10^6^ cell L^−1^). All samples were transferred to the control temperature (22 °C) for 14 days, with a growth rate measurement taken every second day (based on OD measurements) covering the exponential growth period. Growth rate measurements were stopped after 14 days because at this time most cultures had reached the stationary growth phase. The growth rate was calculated using the equation, μ = (ln *Nd *− ln *N*0)/d, where *N*0 and *Nd* represent culture density at the beginning and the end of each test, and d is the duration of the test in days. Number of divisions per day (Div.day^−1^) was calculated as follows: Div.day^−1^ = μ/ln2, while generation time (Gen.t) was calculated according to: Gen.t = 1/Div.day^−1^.

TRNs were completed to determine the optimal and lethal temperature for growth for both ambient and heat-adapted M11 to see if two years of adaptation to increased temperature resulted in a shift in its lethal temperature. Both heat-adapted M11 and ambient-adapted M11 were grown at a range of temperatures from 20 to 40 °C, with intervals of 2 °C. The growth rate was calculated using the same method as above.

### Primer design and optimization PCR

The primers for qPCR were designed with FastPCR software^78^ using In silico Primer search option and targeted cyanobacterial sequences obtained from GenBank as the input. The specificity of the primers was checked by in silico PCR option from the same software and Primer-BLAST^79^. Primers for the reference *rnp*B gene was based on the bibliography search^80,81^. For each primer pair, the annealing temperature (Ta) was optimised through gradient PCR, in reaction mixtures (25 μl) containing: 0.4 μM of each primer, 12.5 μl of 2 × GoTaq® Green Master Mix (Promega), 1 μl cDNA and RNase/DNase-free water. The PCR profile was: 5 min at 95 °C followed by 35 cycles of 30 s at 95 °C, 30 s at 8 different temperatures near the Ta determined by the in silico PCR for each primer pair, 30 s at 72 °C and a final elongation step of 3 min at 72 °C. After optimisation, the size of the PCR products was checked by agarose gel electrophoresis performed by standard protocols, using 1 × TAE buffer and optimal Ta was determined for each primer pair (TableS6).

### RNA extraction, cDNA synthesis and RT-qPCR

After each growth rate experiment (completed at Month 0, 8, 16 and 24) for the ambient and heat-adapted M11, the biomass was centrifuged (to remove the supernatant) and harvested for RNA and subsequent cDNA synthesis and RT-qPCR analysis to determine gene expression of seven thermal tolerance genes. For RNA extraction, the TRIzol® Reagent (Zymo Research) was used in combination with phenol–chloroform extraction. RNA was quantified on a NanoDrop ND-1000 spectrophotometer (Thermo Scientific) and samples were further treated with TURBO DNase treatment (Thermo Scientific) to remove any trace of genomic DNA contamination. For cDNA synthesis, 1 μg of total RNA was transcribed with the iScript™ Reverse Transcription Supermix (Bio-Rad Laboratories) in a final volume of 20 μl, following the manufacturer’s instructions.

The RT-qPCRs were carried out in triplicate on a CFX96 Touch Real-Time PCR Detection System (Bio-Rad) using Eva Green detection chemistry. The reaction mixtures contained the following components: 5 μl 2 × Sso Fast EvaGreen SuperMix, 0.3 μM of the forward and reverse primers, 1 μl cDNA and RNase/DNase-free water to a final volume of 10 μl. The forward and reverse primers of the reference gene (*rnpB*) and target genes (*cya*1, *pnp*, *pyrR*, *clp*C1, *clp*C2 and *sigF*)^25^ were used to determine if these genes were expressed and if there was any difference between ambient and heat-adapted M11 (Table S6). The program consisted of an initial denaturation at 95 °C for 180 s, followed by 44 cycles of 15 s at 95 °C, an annealing step performed at 56 °C for 15 s and an elongation step at 72 °C for 30 s. For reaction specificity assessments, a post-PCR melting curve analysis was performed, in which the temperature ramped between 65 and 95 °C in 0.5 °C increments with subsequent plate readings. Amplification efficiencies of individual runs were checked with the LinRegPCR software. Outliers and samples diverging from the melting curve were omitted from further analysis. Fold change gene expression values of target and reference genes across different experimental conditions were calculated using the comparative Ct method (2^−ΔΔCt^)^82^.

### Land-based mesocosm experiment

The impact of ambient and heat adapted *M. aeruginosa* strain, M11 on natural phytoplankton communities was tested at the Seeon Limnological Station (SLS) of the Ludwig-Maximilians-Universität (LMU) Munich, in Bavaria, Germany during a four-week mesocosm experiment (June-July 2020). Three 1000 L water tanks were used, in which 9 mesocosms were deployed consisting of translucent plastic (LDPE, Polyverpackung GmbH, Trappenkamp, Germany). The three different treatments used were oligotrophic lake water (Lake Klostersee, 48.08°N 11.96°E, TP < 10 mg/L), eutrophic lake water (Lake Bansee, 47.964°N 12.44°E, TP > 30 mg/L) and eutrophic lake water with the addition of bentophos (Dr. Nowak Institute, Ottersberg, Germany). Before the lake water was added to the tanks it was filtered (using a 250 µm gauze), to remove macro- and mesozooplankton. The temperature was not manipulated in any of the tanks (as the expected average July temperature was 23 °C), however a temperature measurement was collected every 15 min using sensors linked to a HOBO MX2202 temperature/light data logger. The three tanks were maintained in this way for 14 days, to allow the planktonic resident communities to acclimate to these conditions, prior to inoculation with *M. aeruginosa* and the addition of the bentophos to treatment 3. Then, the *M. aeruginosa* was inoculated into the lake communities, on June 26th 2020. The use of dialysis bags (10–20 k Dalton pore size; Nadir, Carl Roth, Karlsruhe, Germany), which are permeable for micro- and macro- nutrient, allowed three replicates of both ambient-adapted and heat-adapted *M. aeruginosa* for each treatment. Every enclosure was equipped with three replicate 1.4 L dialysis bags, each filled with water from their respective enclosures. Each dialysis bag was inoculated with strain M11 (initial concentration: 3 × 10^3^ cells mL^−1^) for the controls, with both ambient and heat-adapted M11 being separately inoculated into all three treatments (identical to the methods used for the 2019 mesocosm experiment, as reported in Drugă et al.^22^. In total, the experiment consisted of 36 dialysis bags distributed into 9 enclosures, including the controls (dialysis bags with no added *M. aeruginosa* cells).

### Chlorophyll-*a* concentration measurements

For the mesocosm experiment, chlorophyll *a* concentrations (µgL^−1^) were estimated in vivo using an AlgaeLabAnalyser device (bbe Moldaenke GmbH, Schwentinental, Germany) once a week, for a total of four weeks. The AlgaeLabAnalyser uses specific wavelengths to provide data on phytoplankton community composition based for four defined functional groups of total chlorophyll *a* concentrations. Four different major algal groups were identified: chlorophytes, cryptophytes, cyanobacteria and a brown group, which consists mainly of genera that have additional pigments that absorb in the yellow/orange wavelength range (diatoms, cryophytes, dinoflagellates).

### Nutrients

Nutrient concentrations of nitrite (NO_2_), silicate (SiO_2_), particulate phosphorus (PO_4_-P), nitrate (NO_3_^−^) and total phosphorus (TP) concentrations were collected at the end of the four-week experiment for all treatments.

### DNA analysis

The phytoplankton community composition of each dialysis bag was analysed after the four week mesocosm experiment using DNA metabarcoding. Firstly, the water from each of the three replicates was combined (totalling approx. 3 l), and then centrifuged to remove the excess water. Total DNA was isolated using a E.Z.N.A.® Soil DNA Kit (Omega Bio-tek, Norcross, GA, USA), following the manufacturer’s instructions. The DNA concentration and quality were assessed using a NanoDrop™ 2000 Spectrophotometer (Waltham, MA, USA). An aliquot of the small ribosomal DNA (rDNA) subunit (16S for prokaryotes and 18S for eukaryotes) was then amplified using PCR with primers covering a wide range of planktonic taxa^83,84^. These primers allow for the amplification of a 450 bp DNA fragment within the 16S gene in prokaryotes, and a 650 bp long fragment from the V4 and V5 regions in the 18S gene in eukaryotes (Table S6). DNA sequencing was completed at the LMU Munich. In summary, 5 μL from each PCR reaction were pooled into separate 16S and 18S pools, purified with 0.8 × SPRI beads according to a standard protocol and eluted in 200 μL 10 mM Tris pH7.5. Libraries were constructed using the NEBNext UltraTMII DNA library preparation kit, and sequencing was done with a HiSeq 1500 system (Illumina, San Diego, CA, USA).

Following DNA sequencing, base calling and run demultiplexing were completed using the BaseSpace service (Illumina, San Diego, CA, USA) meters. The pair-end reads were joined in QIIME, and the quality filtration, dereplication and singleton removal was performed using Usearch v8. Both de novo and reference chimera checking were performed in Usearch v8, and using the last version of the Greengenes database (‘13_8’) as a reference^85^. The taxonomy was assigned for the representative OTUs in QIIME using the SSU/LSU 132 SILVA database^86^. The taxonomy was added to the OTU table with the biom-format package, and the mitochondrial and plastid sequences were filtered out of the final OTU table. Rarefaction was performed on 32,584 sequences, followed by alpha- and beta-diversity estimation in QIIME^87,88^. Visualisation of beta-diversity was performed using CANOCO 5 and R (Version 4.1.2).

### Statistics (Data Analysis)

One-way repeated measures ANOVAs (on chlorophyll *a* concentrations) were completed for both ambient- and heat- adapted M11 together and for each version separately using the factor treatment. Prior to analysis the normality, the identification of outliers and assumption of sphericity were checked using a Shapiro Wilk’s test, “identifiy_outliers” function and Mauchly’s test of sphericity respectively. For the repeated measures ANOVAs, the control treatments were excluded as they contained no additional *M. aeruginosa* (and would distort the correlations). Post-hoc analysis included mean separation tests for the multiple comparisons (using Tukey-adjusted comparison) and least square means for the main effects. The analysis was completed using R (version 4.1.2).

Principal coordinate analysis (PCoA) was completed using the Weighted Unifrac of Bray–Curtis distances to determine the differences between treatments. This analysis was completed using the calibrate package in R (version 4.1.2). Principal component analysis (PCA) was conducted on the 16S and 18S metabarcoding data to summarise any groupings between the ambient and heat- adapted M11 and the treatments in an attempt to explain which species could describe these differences. It was completed at phylum and order level for both 16S and 18S genes using Canoco 5. Multiple alpha diversity indexes (Table S5) were used to estimate the diversity of the communities at the end of the experiment.

## Supplementary Information

Supplementary Information.

## Data Availability

All data generated or analyzed during this study are included in this published article (and its Supplementary Material).
