# Supplementary material for: The combined impact of low temperatures and shifting phosphorus availability on the competitive ability of cyanobacteria
Source: Sci Rep. 2022 Sep 30;12:16409. doi: 10.1038/s41598-022-20580-2 (PMC9525609; doi:10.1038/s41598-022-20580-2)
Supplement: Supplementary file 1 — Supplementary Information. [file 41598_2022_20580_MOESM1_ESM.docx]

**Supplementary Information**


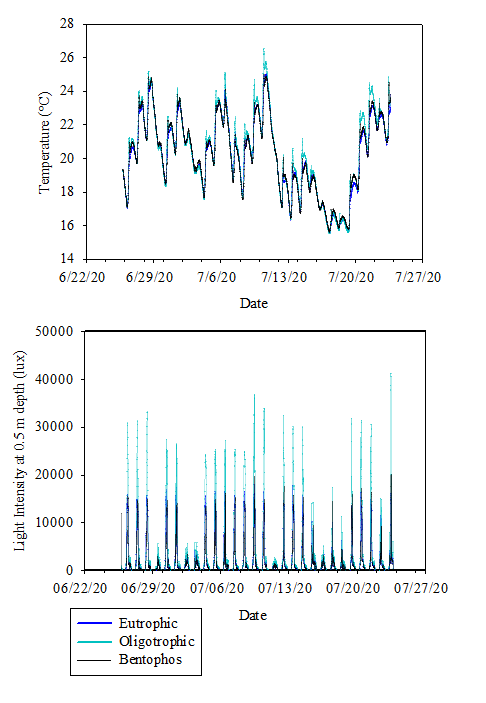


A

B

Figure S1: A) The mean temperature and B) the mean light intensity for each treatment over the four-week experiment

Figure S2: DNA Metabarcoding results to the family level for the 16S gene for prokaryotes collected at the end of the four-week experiment

Figure S3: DNA Metabarcoding results to the family level for the 18S gene for eukaryotes collected at the end of the four-week experiment

Figure S4: PCA biplot of A) 16S to phylum level and B) 18S to order level (due to the dominance of the SAR supergroup) showing both samples and species. T1_CF = Oligotrophic_Control, T1_22F = Oligotrophic_M11_22, T1_26F = Oligotrophic_M11_26, T2_CF = Eutrophic_Control, T2_22F = Eutrophic_M11_22, T2_26F = Eutrophic_M11_26, T3_CF = Bentophos_Control, T3_22F = Bentophos_M11_22, T3_26F = Bentophos_M11_26

B

A

PCA1 – 41.61%

PCA2 – 21.12%

PCA1 – 65.24%

PCA2 – 15.89%

| *Table S1: Nitrite (**NO_2_), silicate (SiO_2_), particulate phosphorus (PO_4_-P), nitrate (**NO_3_^-^) and total phosphorus (TP) concentrations collected at the end of the four-week experiment for all treatments* | | |
| --- | --- | --- |
| Treatment | Parameter | Concentrations (µg L^-1^) |
| Oligotrophic | NO_2_ | 9.16 |
| Oligotrophic | SiO_2_ | 21.14 |
| Oligotrophic | PO_4_-P | 2.47 |
| Oligotrophic | NO_3_^-^ | 298.23 |
| Oligotrophic | TP | 10.77 |
| Eutrophic | NO_2_ | 14.41 |
| Eutrophic | SiO_2_ | 79.71 |
| Eutrophic | PO_4_-P | 4.20 |
| Eutrophic | NO_3_ | 677.61 |
| Eutrophic | TP | 35.41 |
| Eutrophic+Bentophos | NO_2_ | 16.55 |
| Eutrophic+Bentophos | SiO_2_ | 97.49 |
| Eutrophic+Bentophos | PO_4_-P | 2.87 |
| Eutrophic+Bentophos | NO_3_ | 732.01 |
| Eutrophic+Bentophos | TP | 19.18 |

| *Table S2: Repeated measures ANOVA results including the F value, P value and the degrees of freedom based on the chlorophyll a concentrations* | | |
| --- | --- | --- |
| Group Comparison | P value | Results |
| Eutrophic~Oligotrophic | <0.001* | F=202.9, DF=2 |
| Bentophos~Oligotrophic | 0.002* |  |
| Bentophos~Eutrophic | <0.001* |  |
| Week2~Week1 | 0.014* | F=16.67, DF=3 |
| Week 3~Week1 | <0.001* |  |
| Week4~Week1 | <0.001* |  |
| Week3~Week2 | 0.133 |  |
| Week4~Week2 | 0.002* |  |
| Week4~Week3 | 0.511 |  |
| M11_22~Control | 0.858 | F=0.182, DF=2 |
| M11_26~Control | 0.864 |  |
| M11_26~M11_22 | 0.999 |  |

| Table S3: DNA Metabarcoding results to the family level for the 16S gene for prokaryotes collected at the end of the four-week experiment. The numbers represent the percentage of each family in the total pool. | | | | | | | | | |
| --- | --- | --- | --- | --- | --- | --- | --- | --- | --- |
| Family | Eutrophic_M11_22 | Eutrophic_M11_26 | Eutrophic_Control | Bentophos_M11_22 | Bentophos_M11_26 | Bentophos_Control | Oligotrophic_M11_22 | Oligotrophic_M11_26 | Oligotrophic_Control |
| Solibacteraceae | 0.2 | 0.1 | 0.2 | 0.1 | 0.1 | 0.1 | 0.1 | 0.1 | 0.3 |
| Ilumatobacteraceae | 0 | 0 | 0 | 0 | 0 | 0 | 0 | 0 | 0 |
| Mycobacteriaceae | 0.4 | 0.1 | 0 | 0.4 | 0.5 | 0.4 | 0 | 0.2 | 0 |
| Sporichthyaceae | 4.8 | 15.4 | 0.7 | 3.4 | 5.1 | 4.1 | 1 | 2.6 | 1.1 |
| Frankiales_Uncultered | 0 | 0 | 0 | 0 | 0 | 0 | 0 | 0 | 0 |
| Frankiales_Other | 0 | 0 | 0 | 0 | 0 | 0.1 | 0 | 0 | 0 |
| Microbacteriaceae | 0.5 | 2.3 | 0.3 | 0.3 | 0.8 | 0.5 | 0.3 | 0.3 | 0.2 |
| PeM15_uncultured_bacterium | 0.1 | 0.1 | 0 | 0 | 0.1 | 0 | 0 | 0 | 0 |
| Nocardioidaceae | 0 | 0 | 0 | 0 | 0 | 0 | 0 | 0 | 0 |
| Armatimonadaceae | 0.1 | 0.1 | 0.2 | 0.1 | 0.1 | 0.1 | 0.4 | 0.1 | 0.2 |
| Fimbriimonadaceae | 1.1 | 0.9 | 3.7 | 1.4 | 1.4 | 1.2 | 2.2 | 2.2 | 2 |
| BRC1 | 0.1 | 0.1 | 0.1 | 0.1 | 0.1 | 0.1 | 0.1 | 0.1 | 0.2 |
| Flaviramulus_spp. | 0 | 0 | 0 | 0 | 0 | 0 | 0 | 0 | 0 |
| BRC1_1 | 0 | 0 | 0 | 0 | 0 | 0 | 0 | 0 | 0 |
| BRC1_2 | 0.2 | 0.1 | 0 | 0.1 | 0.1 | 0.1 | 0.1 | 0.1 | 0.1 |
| Caenarcaniphilales | 0.1 | 0.1 | 0 | 0 | 0 | 0 | 0 | 0.1 | 0 |
| Caenarcaniphilales_uncultured bacterium | 0 | 0 | 0.1 | 0 | 0 | 0 | 0 | 0 | 0 |
| Obscuribacterales_uncultured bacterium | 0 | 0 | 0 | 0 | 0 | 0 | 0 | 0 | 0 |
| Vampirovibrionales | 0.1 | 0 | 0 | 0 | 0 | 0 | 0 | 0 | 0 |
| Leptolyngbyaceae | 0 | 0 | 0.1 | 0 | 0 | 0 | 0.1 | 0 | 0.3 |
| Chroococcidiopsaceae | 0 | 0 | 0 | 0 | 0 | 0 | 0 | 0 | 0 |
| Microcystaceae | 0 | 0 | 0 | 0 | 0 | 0.1 | 0 | 0 | 0.1 |
| Nostocaceae | 0 | 0 | 0.1 | 0 | 0 | 0 | 0.1 | 0 | 0.1 |
| Oxyphotobacteria Incertae Sedis | 0 | 0 | 0 | 0 | 0 | 0 | 0.1 | 0 | 0.2 |
| Pseudanabaenaceae | 1.3 | 1 | 3.5 | 1.2 | 0.9 | 1 | 3.6 | 0.8 | 5 |
| Rhabdogloea smithii | 0 | 0 | 0 | 0 | 0 | 0 | 0 | 0 | 0 |
| Cyanobiaceae | 3.8 | 3.2 | 0.5 | 2 | 3.1 | 2.4 | 0.5 | 2.7 | 0.9 |
| Bacteroidetes | 0.2 | 0.2 | 0.5 | 0.4 | 0.9 | 0.3 | 0.3 | 0.5 | 0.9 |
| Chitinophagaceae | 0.3 | 0.2 | 0.3 | 0.4 | 0.5 | 0.4 | 0.3 | 0.2 | 0.4 |
| Saprospiraceae | 12.3 | 8.4 | 11.1 | 9.8 | 7.9 | 7.7 | 9.7 | 11.7 | 11.3 |
| Chitinophagale_uncultured | 0.1 | 0.1 | 0 | 0 | 0 | 0.1 | 0 | 0 | 0 |
| Cyclobacteriaceae | 0 | 0 | 0 | 0 | 0.1 | 0 | 0 | 0 | 0 |
| Cytophagaceae | 0 | 0 | 0 | 0 | 0 | 0 | 0 | 0 | 0.1 |
| Microscillaceae | 0.3 | 0.3 | 0.3 | 0.2 | 0.1 | 0.2 | 0.2 | 0.1 | 0.3 |
| Spirosomaceae | 1.8 | 1.7 | 0.8 | 3.2 | 3.5 | 3.1 | 0.8 | 1.1 | 1.6 |
| Crocinitomicaceae | 0.1 | 0.1 | 1.1 | 0.1 | 0.2 | 0.1 | 1 | 0.1 | 0.5 |
| Flavobacteriaceae | 5.9 | 3.6 | 1 | 3.1 | 3.2 | 2.2 | 0.6 | 2.9 | 1 |
| NS9 marine group | 0 | 0 | 0.2 | 0 | 0.1 | 0.1 | 0.2 | 0 | 0.2 |
| Weeksellaceae | 0 | 0 | 0.1 | 0 | 0 | 0 | 0 | 0 | 0 |
| Bacteroidia | 0.1 | 0.1 | 0.3 | 0 | 0 | 0 | 0.3 | 0 | 0.4 |
| Sphingobacteriales | 0.1 | 0.1 | 0 | 0.1 | 0 | 0.1 | 0 | 0 | 0.1 |
| Sphingobacteriales_KD3-93 | 0 | 0 | 0 | 0 | 0 | 0 | 0 | 0 | 0.1 |
| Sphingobacteriales_LiUU-11-161 | 0.4 | 0.3 | 0.7 | 0.1 | 0.2 | 0 | 0.4 | 0.4 | 0.6 |
| Sphingobacteriales_NS11-12 marine group | 1.7 | 1.2 | 2.1 | 1.8 | 1.4 | 1.5 | 1.5 | 1 | 1.5 |
| Sphingobacteriaceae | 0.1 | 0.1 | 0 | 0 | 0.3 | 0.1 | 0 | 0.1 | 0 |
| Sphingobacteriales_env.OPS 17 | 0 | 0 | 0.3 | 0 | 0.1 | 0 | 0.1 | 0 | 0.4 |
| Ignavibacteria | 0.1 | 0.1 | 0.1 | 0.1 | 0.1 | 0.1 | 0.1 | 0.1 | 0.2 |
| Parachlamydiaceae | 0 | 0 | 0 | 0 | 0 | 0 | 0 | 0 | 0 |
| Simkaniaceae | 0 | 0 | 0 | 0 | 0 | 0 | 0 | 0 | 0 |
| Roseiflexaceae | 0.2 | 0.4 | 0 | 0.2 | 1 | 0.1 | 0 | 0.3 | 0 |
| Vermiphilaceae | 0 | 0 | 0 | 0.1 | 0 | 0.1 | 0 | 0.1 | 0 |
| Bacillales | 0 | 0 | 0 | 0 | 0 | 0 | 0.1 | 0 | 0 |
| Gemmatimonadaceae | 0 | 0 | 0 | 0 | 0.1 | 0 | 0 | 0 | 0 |
| Hydrogenedensaceae | 0 | 0 | 0 | 0 | 0 | 0 | 0 | 0 | 0 |
| Magasanikbacteria | 0 | 0 | 0 | 0.2 | 0 | 0.1 | 0 | 0 | 0 |
| Candidatus Peregrinibacteria | 1.2 | 0.5 | 0.6 | 0.5 | 0.3 | 0.2 | 1 | 0.5 | 0.5 |
| Candidatus Peregrinibacteria_Other | 0.1 | 0.1 | 0.1 | 0.1 | 0.1 | 0 | 0 | 0.1 | 0.1 |
| Candidatus Kaiserbacteria | 0.2 | 0.4 | 0 | 0 | 0.1 | 0.1 | 0 | 0.1 | 0 |
| Candidatus Kaiserbacteria_Other | 0.2 | 0.1 | 0 | 1.4 | 2 | 0.8 | 0 | 0.1 | 0 |
| Candidatus Nomurabacteria | 0.3 | 0.1 | 0 | 0 | 0 | 0 | 0 | 0.2 | 0.1 |
| Candidatus Yonathbacteria | 0 | 0 | 0 | 0 | 0 | 0 | 0 | 0 | 0 |
| Parcubacteria | 0 | 0 | 0.5 | 0 | 0 | 0 | 1 | 0 | 0.3 |
| Parcubacteria_Other | 0.1 | 0 | 0 | 0 | 0.1 | 0 | 0 | 0 | 0 |
| Planctomycetes | 0.2 | 0.1 | 0.2 | 0.2 | 0.3 | 0.3 | 0.3 | 0.2 | 0.3 |
| Phycisphaeraceae | 0.7 | 0.6 | 0.8 | 0.9 | 1.3 | 0.9 | 1.1 | 0.8 | 0.9 |
| Tepidisphaerales | 0.3 | 0.2 | 0.1 | 0.1 | 0.2 | 0.2 | 0.3 | 0.1 | 0.5 |
| Gemmataceae | 4.3 | 3.5 | 1.2 | 2.4 | 2.8 | 3.3 | 2 | 4.6 | 1.8 |
| Isosphaeraceae | 0.3 | 0.2 | 0.2 | 0.3 | 0.4 | 0.4 | 0.2 | 0.3 | 0.3 |
| Pirellulaceae | 3.4 | 3.2 | 14.8 | 3 | 2.6 | 3 | 15.1 | 2.8 | 10.4 |
| Rubinisphaeraceae | 0.1 | 0.1 | 0.1 | 0.4 | 0.4 | 0.5 | 0.1 | 0.2 | 0.2 |
| Schlesneriaceae | 0.7 | 0.6 | 3.2 | 1.2 | 1.1 | 1 | 3.1 | 0.8 | 4 |
| Planctomycetales | 1.7 | 1.8 | 2.3 | 1 | 1.4 | 1.1 | 3.8 | 2.1 | 4.9 |
| Acetobacteraceae | 2.6 | 1.7 | 2.8 | 3.1 | 6.4 | 2.8 | 1.9 | 2.2 | 2.4 |
| Azospirillaceae | 0 | 0 | 0.1 | 0 | 0 | 0 | 0.2 | 0.1 | 0.1 |
| Caulobacteraceae | 2.3 | 3.2 | 4.1 | 1.2 | 1.5 | 1.2 | 3.1 | 1.5 | 2.5 |
| Hyphomonadaceae | 0.9 | 1 | 2 | 0.7 | 0.9 | 0.8 | 2 | 0.6 | 1.8 |
| Elsteraceae | 3 | 2.6 | 2.5 | 2.2 | 1.4 | 2.2 | 3.5 | 10.8 | 2.2 |
| Elsterales | 0 | 0 | 0 | 0 | 0 | 0 | 0 | 0 | 0.1 |
| Holosporaceae | 0 | 0 | 0.1 | 0 | 0 | 0 | 0.8 | 0 | 0.1 |
| Micavibrionaceae | 0.2 | 0.1 | 0.3 | 0.2 | 0.6 | 0.3 | 0.3 | 0.2 | 0.4 |
| Micavibrionales | 0.8 | 1.4 | 0.3 | 0.8 | 1.1 | 1 | 0.4 | 0.8 | 0.4 |
| Paracaedibacteraceae | 0.6 | 0.5 | 0 | 0.5 | 0.7 | 0.4 | 0 | 0.7 | 0 |
| Reyranellaceae | 0.1 | 0 | 0.3 | 0 | 0 | 0 | 0.5 | 0 | 0.4 |
| Rhizobiales | 0.1 | 0.1 | 0.1 | 0.1 | 0.1 | 0.1 | 0.1 | 0.1 | 0.1 |
| Beijerinckiaceae | 0.3 | 0.3 | 1.1 | 0.1 | 0.1 | 0.1 | 1.1 | 0.2 | 0.8 |
| Devosiaceae | 4.9 | 3.3 | 1 | 7.4 | 6.6 | 5.9 | 0.6 | 6.1 | 0.8 |
| Hyphomicrobiaceae | 0.1 | 0 | 0 | 0 | 0 | 0 | 0.1 | 0 | 0 |
| Rhizobiaceae | 0.5 | 0.5 | 0.9 | 0.8 | 0.8 | 0.9 | 0.8 | 0.3 | 0.6 |
| Rhizobiales Incertae Sedis | 0.1 | 0.1 | 0.1 | 0.1 | 0.1 | 0.1 | 0.1 | 0 | 0.1 |
| Xanthobacteraceae | 0 | 0 | 0.4 | 0 | 0 | 0 | 0.1 | 0 | 0.2 |
| Rhizobiales_Other | 0 | 0 | 0 | 0 | 0 | 0 | 0.1 | 0 | 0.1 |
| Rhodobacteraceae | 2.7 | 2.2 | 5 | 2.1 | 1.8 | 1.7 | 5.2 | 1.7 | 5 |
| Rhodospirillales | 0.1 | 0.1 | 0.1 | 0.5 | 0.8 | 0.5 | 0.1 | 0.1 | 0.1 |
| Midichloriaceae | 0 | 0 | 0 | 0 | 0 | 0 | 0.1 | 0 | 0.2 |
| Rickettsiaceae | 0 | 0 | 0.1 | 0 | 0 | 0 | 0.3 | 0 | 0.3 |
| Rickettsiales | 1 | 0.8 | 1.4 | 0.8 | 1.1 | 0.8 | 0.9 | 0.8 | 0.8 |
| Rickettsiales_Other | 0 | 0 | 0 | 0.3 | 0.1 | 0 | 0 | 0 | 0 |
| SAR11 clade_Clade III | 0.4 | 2.6 | 0 | 0 | 0.1 | 0 | 0 | 0.4 | 0.1 |
| Sphingomonadaceae | 3.2 | 3 | 4 | 3.9 | 6.3 | 3.7 | 3.1 | 2.3 | 3.1 |
| Alphaproteobacteria | 0 | 0 | 0 | 0 | 0 | 0 | 0 | 0 | 0 |
| Alphaproteobacteria_Other | 0.2 | 0.3 | 0 | 0 | 0.1 | 0 | 0 | 0.1 | 0 |
| Bacteriovoracaceae | 0 | 0 | 0 | 0 | 0 | 0 | 0 | 0 | 0 |
| Bdellovibrionaceae | 0.3 | 0.2 | 1.5 | 0.4 | 0.4 | 0.3 | 1.7 | 0.3 | 1.6 |
| Bradymonadales | 0.1 | 0.1 | 0.4 | 0.5 | 0.1 | 0.1 | 0.4 | 0.2 | 0.2 |
| Myxococcales | 0 | 0 | 0 | 0 | 0 | 0 | 0 | 0 | 0 |
| Phaselicystidaceae | 0 | 0 | 0.2 | 0 | 0 | 0 | 0.1 | 0 | 0.1 |
| Sandaracinaceae | 0.1 | 0 | 0.1 | 0.2 | 0.1 | 0 | 0.1 | 0.1 | 0.1 |
| Myxococcales | 0.1 | 0.1 | 0.2 | 0.1 | 0.1 | 0 | 0.3 | 0.2 | 0.2 |
| Oligoflexales | 0 | 0 | 0 | 0 | 0 | 0 | 0.1 | 0 | 0.1 |
| Oligoflexaceae | 0.7 | 0.4 | 0 | 0 | 0 | 0 | 0 | 0.4 | 0.2 |
| Deltaproteobacteria_SAR324 clade | 0 | 0 | 0 | 0.1 | 0.1 | 0.1 | 0 | 0 | 0 |
| Burkholderiaceae | 3.1 | 4 | 4.1 | 2.6 | 4.3 | 3.2 | 3.5 | 2.9 | 2.7 |
| Chromobacteriaceae | 0 | 0.4 | 0 | 0.1 | 0.2 | 0.1 | 0.1 | 0.1 | 0 |
| Methylophilaceae | 0.5 | 0.5 | 0.2 | 0.8 | 0.6 | 0.8 | 0.1 | 0.4 | 0.3 |
| Betaproteobacteriales | 0 | 0 | 0 | 0 | 0.1 | 0 | 0 | 0 | 0 |
| Betaproteobacteriales | 0 | 0 | 0 | 0 | 0 | 0 | 0 | 0 | 0.1 |
| Cellvibrionaceae | 0 | 0 | 0 | 0 | 0 | 0 | 0 | 0 | 0 |
| Gammaproteobacteria | 0 | 0 | 0 | 0 | 0 | 0 | 0 | 0 | 0 |
| Gammaproteobacteria Incertae Sedis | 0 | 0 | 0 | 0 | 0 | 0 | 0 | 0 | 0 |
| Legionellaceae | 0.1 | 0 | 0 | 0.1 | 0.1 | 0.2 | 0 | 0 | 0 |
| Moraxellaceae | 0.2 | 0.2 | 0.3 | 0.1 | 0.2 | 0.2 | 0.2 | 0.2 | 0.3 |
| Solimonadaceae | 0 | 0 | 0.2 | 0.1 | 0.1 | 0.1 | 0.1 | 0.1 | 0.1 |
| Rhodanobacteraceae | 0 | 0 | 0 | 0 | 0.1 | 0.1 | 0 | 0 | 0 |
| Gammaproteobacteria_Other | 0 | 0 | 0.1 | 0 | 0 | 0 | 0.1 | 0 | 0.1 |
| Leptospiraceae | 0.1 | 0.1 | 0.1 | 0.1 | 0.1 | 0 | 0.1 | 0.1 | 0 |
| Chthoniobacteraceae | 0.5 | 0.6 | 0.1 | 0.3 | 0.3 | 0.3 | 0.2 | 0.4 | 0.3 |
| Terrimicrobiaceae | 0.1 | 0.2 | 0.1 | 0.2 | 0.1 | 0.3 | 0.2 | 0.1 | 0.2 |
| Methylacidiphilaceae | 0.5 | 0.8 | 0.2 | 0.2 | 0.2 | 0.2 | 0.3 | 0.6 | 0.3 |
| Opitutaceae | 0.4 | 0.7 | 0.3 | 0.3 | 0.1 | 0.5 | 0.4 | 1 | 0.3 |
| Pedosphaeraceae | 0.3 | 0.1 | 0.4 | 0.1 | 0.1 | 0.2 | 0.8 | 0.4 | 0.7 |
| Verrucomicrobiales | 0.1 | 0.1 | 0.3 | 0.1 | 0.1 | 0.1 | 0.3 | 0.1 | 0.3 |
| Rubritaleaceae | 0.3 | 0.2 | 0.2 | 0.3 | 0.4 | 0.4 | 0.3 | 0.3 | 0.5 |
| Verrucomicrobiaceae | 12.2 | 7.6 | 7.1 | 9 | 5.3 | 8.6 | 9.9 | 15.3 | 9.8 |
| Unassigned | 5.5 | 7 | 3.6 | 18.7 | 11.2 | 22.7 | 3.1 | 6.6 | 4 |

| Table S4: DNA Metabarcoding results to the family level for the 18S gene for eukaryotes collected at the end of the four-week experiment. The numbers represent the percentage of each family in the total pool. | | | | | | | | | |
| --- | --- | --- | --- | --- | --- | --- | --- | --- | --- |
| Family | Eutrophic_M11_22 | Eutrophic_M11_26 | Eutrophic_Control | Bentophos_M11_22 | Bentophos_M11_26 | Bentophos_Control | Oligotrophic_M11_22 | Oligotrophic_M11_26 | Oligotrophic_Control |
| Discosea | 0 | 0 | 0 | 0.024402 | 0.00338 | 0.027169 | 0 | 0 | 0 |
| Charophyta_Ambiguous_taxa | 0.53072 | 0.779303 | 0.025727 | 0.219619 | 0.226168 | 0.40753 | 0.071867 | 0.459997 | 0.085265 |
| Fagales | 0 | 0.561823 | 0 | 0 | 0.00675 | 0 | 0.06081 | 0 | 0.015503 |
| Charophyta_Other | 0.00281 | 0 | 0.036753 | 0.00349 | 0 | 0 | 0.027641 | 0.00235 | 0.00388 |
| Chlorophyta_Ambiguous_taxa | 2.625519 | 2.514612 | 0.837958 | 1.600084 | 1.741831 | 2.119154 | 0.829233 | 2.572227 | 1.813813 |
| Chlorophyceae | 1.308548 | 0.697748 | 0.569664 | 0.9726 | 1.616932 | 1.358432 | 0.563879 | 0.746321 | 1.193706 |
| Chlamydomonadales | 0.030888 | 0.022654 | 0.029402 | 0.066234 | 0.07764 | 0.050456 | 0.016585 | 0.070408 | 0.015503 |
| Chlorellales | 0.061777 | 0.013592 | 0.014701 | 0.020916 | 0.00338 | 0.011644 | 0.016585 | 0.03051 | 0.00388 |
| Sphaeropleales | 0.221835 | 0.457614 | 0.073505 | 0.233563 | 0.266676 | 0.582185 | 0.09398 | 0.138468 | 0.178281 |
| Chlorophyto_Other | 1.895429 | 3.742467 | 0.768128 | 1.617514 | 1.71145 | 1.533088 | 0.873459 | 1.419888 | 1.077436 |
| Centrohelida | 0.00281 | 0.00906 | 0.00368 | 0 | 0.00338 | 0.00776 | 0.044226 | 0.00704 | 0.120146 |
| Pterocystis_Other | 0.00842 | 0 | 0.011026 | 0.25448 | 0.043883 | 0.073743 | 0 | 0.00469 | 0.00775 |
| Acanthocystidae | 0.508256 | 0.045308 | 0 | 0.038346 | 0.067513 | 0.03105 | 0 | 0.103265 | 0.046508 |
| Centrohelida_H15-6 | 0.00842 | 0.086086 | 0 | 0.00349 | 0.00675 | 0 | 0.00553 | 0.00704 | 0.00775 |
| Heterophryidae | 0.00842 | 0 | 0 | 0 | 0.00338 | 0 | 0 | 0 | 0.019378 |
| Centrohelida | 0.474559 | 0.457614 | 0.194788 | 0.498501 | 0.482717 | 1.19542 | 0.165847 | 0.586731 | 1.085187 |
| Diphylleia rotans | 0.016848 | 0.022654 | 0.033077 | 0.379976 | 0.00338 | 0.225112 | 0.027641 | 0.028163 | 0.00775 |
| Cryptomonas | 0.070201 | 0.466676 | 0.00735 | 0.041832 | 0.047259 | 0.077625 | 0.033169 | 0.06102 | 0.046508 |
| Cryptomonas | 0.030888 | 0.185764 | 0 | 0.013944 | 0.013503 | 0.0621 | 0.00553 | 0.016429 | 0.00775 |
| Rhodomonas | 0.00562 | 0.00453 | 0.00735 | 0 | 0 | 0.00388 | 0.00553 | 0.00939 | 0.00388 |
| Goniomonas_sp. SH-8 | 0.025272 | 0.031716 | 0.00368 | 0.027888 | 0.010127 | 0.027169 | 0 | 0.053979 | 0.00388 |
| Goniomonas_uncultured microeukaryote | 0.25834 | 0.321689 | 0.029402 | 0.278882 | 0.040508 | 0.124199 | 0.038698 | 0.682954 | 0.062011 |
| Goniomonas | 0.106706 | 0.05437 | 0.022052 | 0.177787 | 0.027005 | 0.244518 | 0.016585 | 0.164285 | 0.00775 |
| Kathablepharidae_uncultured freshwater eukaryote | 0.01404 | 0 | 0.00735 | 0.090637 | 0.00675 | 0.104793 | 0.011056 | 0.014082 | 0 |
| Kathablepharidae | 0.047737 | 0.240134 | 0 | 0.142927 | 0.033756 | 0.19018 | 0.016585 | 0.058673 | 0.015503 |
| Haptophyta_uncultured | 0.019656 | 0.00906 | 0 | 0.010458 | 0.023629 | 0.015525 | 0 | 0.014082 | 0.00775 |
| Micronuclearia podoventralis | 0.00562 | 0.036247 | 0.095557 | 0.024402 | 0.027005 | 0.034931 | 0.099508 | 0.023469 | 0.054259 |
| Craspedida | 0.042121 | 0.058901 | 0.022052 | 0.059262 | 0.060762 | 0.097031 | 0.016585 | 0.110305 | 0.034881 |
| Codosigidae | 0.00562 | 0.045308 | 0 | 0 | 0.030381 | 0 | 0.00553 | 0 | 0 |
| Salpingoecidae | 0.044929 | 0.289973 | 0.28667 | 0.083665 | 0.060762 | 0.015525 | 0.033169 | 0.145509 | 0.093016 |
| Dermocystida | 0.831181 | 0.888043 | 0.022052 | 0.41135 | 0.246422 | 0.620997 | 0.116093 | 0.844892 | 0.170529 |
| Gastrotricha | 0.016848 | 0 | 0.033077 | 0.345116 | 0.145153 | 0.53561 | 0.016585 | 0.023469 | 0.00775 |
| Bdelloidea | 0 | 0 | 0.00735 | 0 | 0 | 0 | 0 | 0.037551 | 0 |
| Monogononta | 2.617095 | 3.080966 | 0.562314 | 0.711148 | 1.654064 | 2.592664 | 1.675051 | 2.398554 | 1.802186 |
| Copepoda | 4.021116 | 5.119841 | 21.84939 | 13.04469 | 24.86835 | 0.729672 | 19.54226 | 3.112019 | 17.64204 |
| Phyllopoda | 0.073009 | 0.244665 | 0.124959 | 0.027888 | 0.911423 | 0.419173 | 0.044226 | 0.035204 | 0.135648 |
| Fungi_Ambiguous_taxa | 0.123554 | 0.222011 | 0.033077 | 0.139441 | 0.435458 | 0.100912 | 0.044226 | 0.068061 | 0.069762 |
| Cryptomycota | 1.471414 | 0.729464 | 0.316072 | 3.203653 | 3.139346 | 4.513875 | 0.226657 | 1.466826 | 0.523215 |
| Fungi_LKM15 | 0.508256 | 0.16311 | 0.084531 | 0.498501 | 0.560356 | 0.706385 | 0.055282 | 0.455303 | 0.096892 |
| Blastocladiales | 0.025272 | 0 | 0.00368 | 0.010458 | 0.364569 | 0.00776 | 0 | 0.018775 | 0.038757 |
| Rhizophydiales | 0.030888 | 0.031716 | 0.00368 | 0.06972 | 0.037132 | 0.065981 | 0.011056 | 0.035204 | 0.00388 |
| Incertae Sedis | 0.011232 | 0 | 0.00368 | 0.00697 | 0.016878 | 0.00388 | 0 | 0.00235 | 0 |
| Rhizophydiaceae | 0.019656 | 0.018123 | 0.036753 | 0.013944 | 0.020254 | 0.034931 | 0.088452 | 0.016429 | 0.058135 |
| Filobasidiaceae | 0.00842 | 0 | 0 | 0 | 0.00338 | 0.00388 | 0 | 0.00469 | 0.00388 |
| Fungi_Other | 0.050545 | 0.072493 | 0.044103 | 0.115039 | 0.064137 | 0.128081 | 0.011056 | 0.058673 | 0.081389 |
| Eugregarinorida | 0 | 0 | 0 | 0 | 0 | 0.03105 | 0 | 0.00469 | 0 |
| Ciliophora_Ambiguous_taxa | 0.294844 | 0.507453 | 0.106582 | 0.948198 | 1.99838 | 2.588783 | 0.24877 | 0.283978 | 0.096892 |
| Litostomatea | 0.146018 | 0.480268 | 0.033077 | 0.219619 | 0.138401 | 0.217349 | 0.06081 | 0.100918 | 0.054259 |
| Spirotrichea | 0.12917 | 0.077024 | 0.036753 | 0.317228 | 0.330813 | 0.640404 | 0.00553 | 0.077448 | 0.046508 |
| Euplotia | 0.016848 | 0.031716 | 0 | 0.010458 | 0.016878 | 0.077625 | 0.022113 | 0.014082 | 0.108519 |
| Haptoria | 1.825227 | 1.436274 | 0.378551 | 2.551767 | 2.52498 | 2.759558 | 1.370999 | 2.445493 | 1.340981 |
| Hypotrichia | 0.067393 | 0.067962 | 0.117608 | 0.01743 | 0.020254 | 0.034931 | 0.862403 | 0.063367 | 0.100767 |
| Oligohymenophorea | 0.463327 | 0.992252 | 0.257268 | 1.310744 | 0.398326 | 1.470988 | 0.43673 | 0.718158 | 0.182156 |
| Oligotrichia | 0.053353 | 0.058901 | 0.044103 | 0.05229 | 0.07764 | 0.03105 | 0.038698 | 0.011735 | 0.08914 |
| Phyllopharyngea | 0 | 0 | 0.194788 | 0.027888 | 0.00338 | 0 | 0 | 0 | 0 |
| Prostomatea | 1.389981 | 3.171583 | 0.154361 | 1.2968 | 2.086146 | 3.240831 | 0.414617 | 2.586308 | 0.422448 |
| Cyrtolophosidida | 0.15725 | 0.403244 | 0.282995 | 0.68326 | 0.324062 | 0.256161 | 0.486484 | 0.246427 | 0.379816 |
| Hymenostomatia | 0.280804 | 0.10874 | 0.014701 | 0.00349 | 0.249797 | 0.0621 | 0.679971 | 0.021122 | 1.290598 |
| Peniculia | 0.064585 | 0.013592 | 0.062479 | 0.223105 | 0.037132 | 0.015525 | 0.016585 | 0.016429 | 0.00388 |
| Peritrichia | 1.797147 | 3.461556 | 0.246242 | 0.160357 | 0.378072 | 0.34543 | 0.773951 | 2.781103 | 0.651112 |
| Scuticociliatia | 1.521959 | 0.842735 | 0.290345 | 3.262916 | 1.029571 | 5.938288 | 0.315109 | 2.452534 | 0.263546 |
| Ciliophora_Other | 0.056161 | 0.05437 | 0.06983 | 0.226591 | 0.236295 | 0.768484 | 0.326165 | 0.06102 | 0.306178 |
| Gymnodinium clade | 0.087049 | 1.96185 | 0.011026 | 0.153385 | 0.030381 | 0.240637 | 0.226657 | 0.089183 | 0.08914 |
| Peridiniales | 0.345389 | 1.123646 | 0.084531 | 0.533361 | 0.651499 | 0.492917 | 0.127149 | 0.309794 | 0.131773 |
| Suessiaceae | 0.070201 | 0.117802 | 0.00368 | 0.090637 | 0.027005 | 0.046575 | 0.022113 | 0.168978 | 0.015503 |
| Thoracosphaeraceae | 0.00281 | 0.00906 | 0 | 0.01743 | 0.023629 | 0.027169 | 0 | 0.00469 | 0 |
| Dinoflagellata_Other | 0.00842 | 0 | 0 | 0 | 0 | 0 | 0.00553 | 0 | 0.046508 |
| Colpodellida | 0.022464 | 0.031716 | 0 | 0.01743 | 0.070888 | 0.104793 | 0 | 0.025816 | 0.023254 |
| Cercomonadidae | 0.019656 | 0.027185 | 0.058804 | 0.024402 | 0.016878 | 0.038812 | 0.077395 | 0.056326 | 0.050384 |
| Clathrulinidae | 0.134786 | 0.086086 | 0.014701 | 0.25448 | 0.081015 | 0.294974 | 0.049754 | 0.21357 | 0.019378 |
| Glissomonadida | 0.061777 | 0.104209 | 0.018376 | 0.101095 | 0.114772 | 0.09315 | 0.027641 | 0.105612 | 0.031005 |
| Incertae Sedis | 0.056161 | 0.031716 | 0.073505 | 0.031374 | 0.020254 | 0.027169 | 0.105036 | 0.03051 | 0.073638 |
| Cercozoa_Gran-3 | 0.044929 | 0.05437 | 0.00735 | 0.010458 | 0.047259 | 0.015525 | 0.033169 | 0.058673 | 0.031005 |
| Cercozoa_Gran-4 | 0.233068 | 0.471207 | 0.066155 | 1.310744 | 0.101269 | 0.139724 | 0.038698 | 0.394283 | 0.054259 |
| Cercozoa_Gran-5 | 0.053353 | 0.022654 | 0.051454 | 0.024402 | 0.05401 | 0.011644 | 0.077395 | 0.011735 | 0.228664 |
| Vampyrellidae | 4.470403 | 1.445336 | 0.389577 | 0.216133 | 0.486092 | 0.155249 | 0.204544 | 0.863667 | 1.585148 |
| Cercozoa_uncultured | 0.058969 | 0.013592 | 0.00368 | 0 | 0.00338 | 0.00388 | 0 | 0.00704 | 0.00775 |
| Incertae Sedis | 0.216219 | 0.303566 | 0.029402 | 0.024402 | 0.810154 | 0.011644 | 0.027641 | 0.107958 | 0.093016 |
| Euglyphida | 0.011232 | 0.013592 | 0.014701 | 0.03486 | 0.010127 | 0.03105 | 0.027641 | 0.00469 | 0.034881 |
| Cercozoa_Other | 0.277996 | 0.013592 | 0.025727 | 0.00697 | 0.904672 | 0 | 0.011056 | 0.00939 | 0.104643 |
| Bicosoeca | 0 | 0.00453 | 0.036753 | 0 | 0.016878 | 0.00388 | 0.038698 | 0 | 0.108519 |
| Bicosoecida_LG08-10 | 0.00562 | 0.013592 | 0 | 0 | 0 | 0 | 0 | 0.011735 | 0 |
| Bicosoecida_uncultured | 0.011232 | 0 | 0.00368 | 0.03486 | 0 | 0 | 0 | 0 | 0 |
| Bicosoecida_Other | 0.050545 | 0.045308 | 0.183763 | 0.03486 | 0.057386 | 0.023287 | 0.359334 | 0.00939 | 0.434075 |
| Stramenopiles_MAST-12C | 0.266764 | 1.236917 | 0.338123 | 0.139441 | 0.249797 | 0.291093 | 0.519653 | 0.143162 | 0.573599 |
| Ochrophyta_Ambiguous_taxa | 1.086712 | 1.173486 | 3.884744 | 1.987032 | 1.286119 | 3.256356 | 8.944663 | 1.283766 | 2.7556 |
| Chrysophyceae | 7.477816 | 7.951611 | 24.66463 | 12.49739 | 8.746287 | 10.40171 | 22.47222 | 8.247084 | 18.01411 |
| Dictyochophyceae | 0.699203 | 0.607132 | 0.521886 | 4.55623 | 1.741831 | 2.068698 | 0.09398 | 0.485813 | 0.228664 |
| Chromulinales | 35.95698 | 29.93068 | 33.9079 | 13.64429 | 9.401161 | 7.847856 | 28.95682 | 30.52641 | 33.48578 |
| Eustigmatales | 0.019656 | 0.013592 | 0.00735 | 0 | 0.00675 | 0.015525 | 0 | 0.00939 | 0 |
| Hibberdiales | 0.011232 | 0.00453 | 0 | 0.00697 | 0.023629 | 0.034931 | 0 | 0.014082 | 0.019378 |
| Ochromonadales | 7.45816 | 6.474559 | 3.870043 | 13.68961 | 12.48987 | 14.28682 | 3.372215 | 16.52468 | 4.763197 |
| Pedinellales | 0.030888 | 0 | 0.00368 | 0.020916 | 0.151904 | 0.042694 | 0.00553 | 0.00704 | 0.00775 |
| Bacillariophyceae | 0.648658 | 0.294504 | 0.150685 | 1.376978 | 0.830408 | 1.630118 | 0.077395 | 0.607853 | 0.166654 |
| Mediophyceae | 5.939009 | 5.156087 | 0.882061 | 7.808687 | 5.387524 | 9.862216 | 0.890044 | 5.84853 | 1.096814 |
| Ochrophyta_Other | 1.412445 | 0.534638 | 0.518211 | 1.934742 | 1.900486 | 2.728508 | 0.30958 | 1.901007 | 0.49221 |
| Peronosporomycetes_Ambiguous_taxa | 0.146018 | 0.65244 | 0.029402 | 0.125497 | 0.283554 | 0.027169 | 0.121621 | 0.072755 | 0.379816 |
| Peronosporomycetes_Haptoglossa | 0.016848 | 0.00906 | 0 | 0.00349 | 0.00338 | 0.054337 | 0.011056 | 0.016429 | 0.011627 |
| Peronosporomycetes_Pythium | 0.089857 | 0.117802 | 0.033077 | 0.097609 | 0.15528 | 0.147487 | 0.022113 | 0.098571 | 0.038757 |
| Peronosporomycetes_Other | 0.255532 | 0.208418 | 0.036753 | 0.240535 | 0.398326 | 0.194062 | 0.016585 | 0.150203 | 0.08914 |
| Unassigned | 5.787375 | 6.306919 | 1.580359 | 2.154361 | 4.452471 | 7.785756 | 1.757975 | 3.586097 | 2.724595 |

| *Table S5: Alpha Diversity Indexes (including Chao1, PD Whole Tree, Simpson and the Shannon-Wiener Index) based on the 16S and 18S gene calculated using the total number of reads* | | | | | | | |
| --- | --- | --- | --- | --- | --- | --- | --- |
| Sample | Chao 1 | | PD Whole Tree | Simpson | | Shannon−Wiener index | |
|  | 16S | 18S | 16S | 16S | 18S | 16S | 18S |
| Eutrophic_Control | 428.029 | 262.000 | 33.915 | 0.973 | 0.793 | 6.629 | 3.313 |
| Eutrophic_M11_22 | 431.500 | 301.894 | 33.478 | 0.974 | 0.875 | 6.552 | 4.885 |
| Eutrophic_M11_26 | 416.966 | 277.077 | 33.216 | 0.974 | 0.910 | 6.507 | 5.238 |
| Bentophos_Control | 424.636 | 292.333 | 33.467 | 0.949 | 0.952 | 6.115 | 5.567 |
| Bentophos_M11_22 | 418.029 | 266.531 | 32.915 | 0.962 | 0.936 | 6.224 | 5.077 |
| Bentophos_M11_26 | 424.935 | 311.794 | 32.681 | 0.976 | 0.915 | 6.562 | 5.104 |
| Oligotrophic_Control | 415.600 | 277.078 | 34.074 | 0.980 | 0.840 | 6.820 | 4.155 |
| Oligotrophic_M11_22 | 397.500 | 277.667 | 32.936 | 0.975 | 0.835 | 6.669 | 3.763 |
| Oligotrophic_M11_26 | 384.00 | 285.000 | 32.490 | 0.962 | 0.895 | 6.071 | 4.839 |

| *Table S6: List of M. aeruginosa genes including the reference gene (rnpB ^[80]^) and genes used in temperature tolerance ^[25]^. The description of each gene, amplicon size and primer sequence are also included.*  **Source ^[83]^, ** ^[84]^* | | | | |
| --- | --- | --- | --- | --- |
| Gene | Descriptions | Primer Sequence | | Amplicon Size |
| rnpB | RNA component of ribonuclease P (RNase P) (catalytic subunit, ribozyme), function: cleavage of precursor sequences from the 5 ends of pre-tRNAs | F | 5’-gtggggagcaaggtgg -3’ | 121 pb |
|  |  | R | 5’-cttttacctttgttggaatagag -3’ |  |
| pyrR | Transcriptional antiterminator with minor uracil phosphoribosyltransferase activity, function: regulation of pyrimidine biosynthesis | F | 5’-ccaaaatgcccctagatgtcacggg-3’ | 267 pb |
|  |  | R | 5’-tgattaattccaccccatctttgccg-3’ |  |
| cya1 | Adenylate cyclase 1, function: regulation of cellular metabolism by catalyzing the synthesis of a second messenger, cAMP | F | 5'-gacgaaaaagtgctttcttctctgattgg-3’ | 132 pb |
|  |  | R | 5'-ctgttggccgtgaaaccagatgg-3’ |  |
| sigF | RNA polymerase forespore-specific (early) sigma factor SigF, function: transcription of sporulation genes | F | 5’-ttagytcyttcgctattccctatattcg-3’ | 452 pb |
|  |  | R | 5’-taattgttcggcggtttcccg-3’ |  |
| pnp | Polyribonucleotide nucleotidyltransferase, function: involved in mRNA degradation | F | 5’-cagatggatatgaaaatccccg-3’ | 320 pb |
|  |  | R | 5’-atcagtcgtttagctttttcgg-3’ |  |
| nlpD | Murein hydrolase activator, function: lysozyme/metalloendopeptidase, activator of the cell wall hydrolase AmiC | F | 5'-tcaggtgatcccgattcccgttcccac-3’ | 282 pb |
|  |  | R | 5'-ttggggttgtcaatgccgttttccc-3’ |  |
| pyk | Pyruvate kinase, function catabolic enzyme in glycolysis | F | 5'-aaaccgatgttattgctcgatttaccc-3’ | 339 pb |
|  |  | R | 5'-gataattccgccagctttccgc-3’ |  |
| clcpC1 | ATP-dependent Clp protease ATP-binding subunit ClpC1, function: ATP-dependent specificity component of the Clp protease, can perform chaperone functions | F | 5’ ggttacagtcccgttaatatccctttt-3’ | 239 pb |
|  |  | R | 5’- aagcgaccactagcgactttttcccc-3’ |  |
| clcpC2 | Chaperone protein ClpC2, function: molecular chaperone | F | 5’-gccattaaagtaatcatgctcgccc-3’ | 336 pb |
|  |  | R | 5’-ggccacaccttccccttcgc-3’ |  |
| 16S* | RNA component of the 30S subunit of the Prokaryotic ribosome | F | 5’-cct acg ggn ggc wgc ag-3’ | 450 pb |
|  |  | R | 5’-gac tac hvg ggt atc taa tcc-3’ |  |
| 18S** | RNA component of the 40S subunit of the Eukaryotic ribosome | F | 5’-cag ccg cgg taa ttc c-3’ | 650 pb |
|  |  | R | 5’-ccc gtg ttg agt caa att aag c-3’ |  |
